# Supplementary material for: ASH1L-MRG15 methyltransferase deposits H3K4me3 and FACT for damage verification in nucleotide excision repair
Source: Nat Commun. 2023 Jul 1;14:3892. doi: 10.1038/s41467-023-39635-7 (PMC10314917; doi:10.1038/s41467-023-39635-7)
Supplement: Supplementary file 1 — Supplementary Information [file 41467_2023_39635_MOESM1_ESM.pdf]

Supplementary information

ASH1L-MRG15 methyltransferase deposits H3K4me3 and FACT for damage verification in nucleotide excision repair

Corina Maritz[1,3], Reihaneh Khaleghi[1,3], Michelle N. Yancoskie[1,3], Sarah Diethelm[1], Sonja Brülisauer[1], Natalia Santos Ferreira[1], Yang Jiang[2], Shana J. Sturla[2], Hanspeter Naegeli[1]*

[1]Institute of Pharmacology and Toxicology, University of Zurich-Vetsuisse, Zurich, Switzerland
[2]Department of Health Sciences and Technology, ETH Zurich, Zurich, Switzerland.
[3]These authors contributed equally: Corina Maritz, Reihaneh Khaleghi and Michelle N. Yancoskie

*E-mail: hanspeter.naegeli@uzh.ch

a

Targeted gene	Targeted exon	Guide RNA 5'-3'
ASH1L	Exon 2	TGCCATCAGTACTGGCACAT<br>TCGCAAACGGAATCGAGAAA
	Exon 11, 12	TCTAGAACGATTTCGAGCTG<br>GTGATTGACAGTTACCGCAT
XPC	Exon 1	ATGGCTCGGAAACGCGCGGC
	Exon 2	TCAGCGATGGGGATGACCTC
XPA	Exon 1	CGGTGCGGGCGAGTATCGAG
XPF	Exon 1	TGGAACTGCTCGACACTGAC
	Exon 2	CGCTATGAAGTTTACACACA
non-targeted		GTATTACTGATATTGGTGGC

b

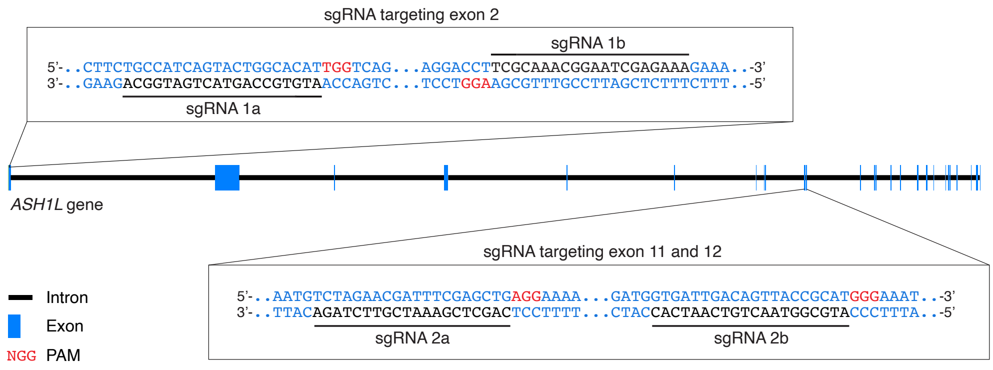

Supplementary Fig. 1. Gene deletions in U2OS cells. The CRISPR-Cas9 (Clustered Regularly Interspaced Short Palindromic Repeats-CRISPR-associated protein 9) system was employed for *ASH1L*, *XPC*, *XPA* and *XPF* gene deletions in human U2OS cells. **a** List of guide RNA sequences employed for gene deletions. **b** Scheme illustrating how the human *ASH1L* gene was targeted for disruption leading to two distinct deletions in exon 2 and exons 11/12. PAM, protospacer adjacent motif; sgRNA, single guide RNA.

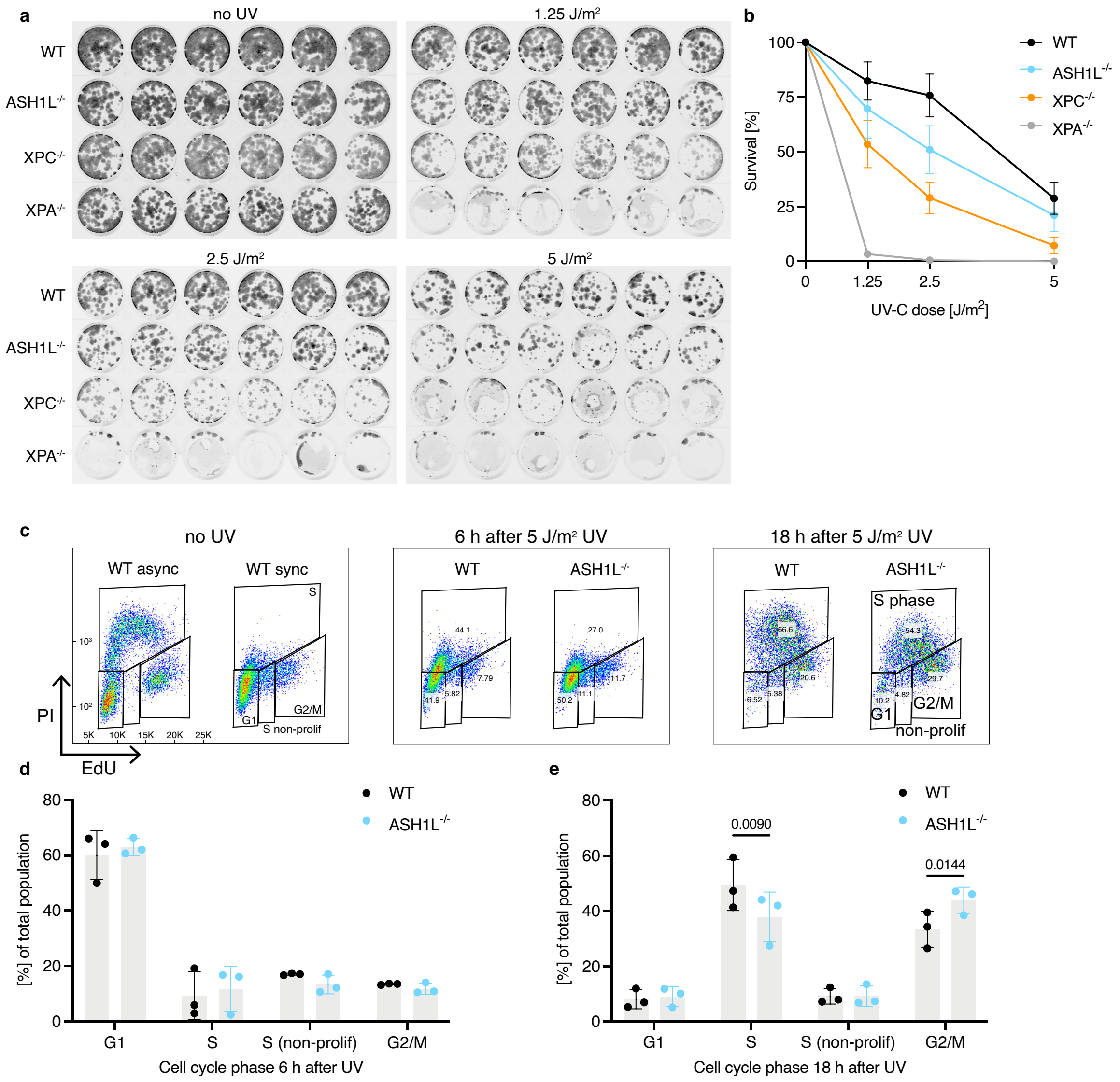

Supplementary Fig. 2. Clonal survival and cell cycle analyses. The *ASH1L* deletion reduces cell survival and perturbs the cell cycle upon UV damage, consistent with impaired UV lesion excision. **a** Wild-type (WT), ASH1L$^{-/-}$, XPC$^{-/-}$ and XPA$^{-/-}$ cells were exposed to different UV doses and left to recover for 7 days in 24-well plates. **b** Quantification of colony survival. Colony numbers are expressed as the percentage of unirradiated controls. Mean values ± SD, n = 3 independent experiment, each with 6 technical replicates. **c** WT and ASH1L$^{-/-}$ cells were UV-treated and released from mimosine-induced G1 arrest 6 or 18 h before flow cytometry. DNA synthesis was monitored by EdU incorporation. **d** Proportion of U2OS cells in the indicated cell cycle phases 6 h after UV irradiation. **e** Proportion of U2OS cells in the indicated cell cycle phases 18 h after UV irradiation. This analysis revealed that, in response to a UV challenge, the *ASH1L* deletion decreases the population of S-phase cells, compensated by more cells arrested in G2-M compared to WT controls. Panels **d** and **e** show mean values ± SD (n = 3, each experiment with 3 technical replicates). Significance was tested by two-way ANOVA.

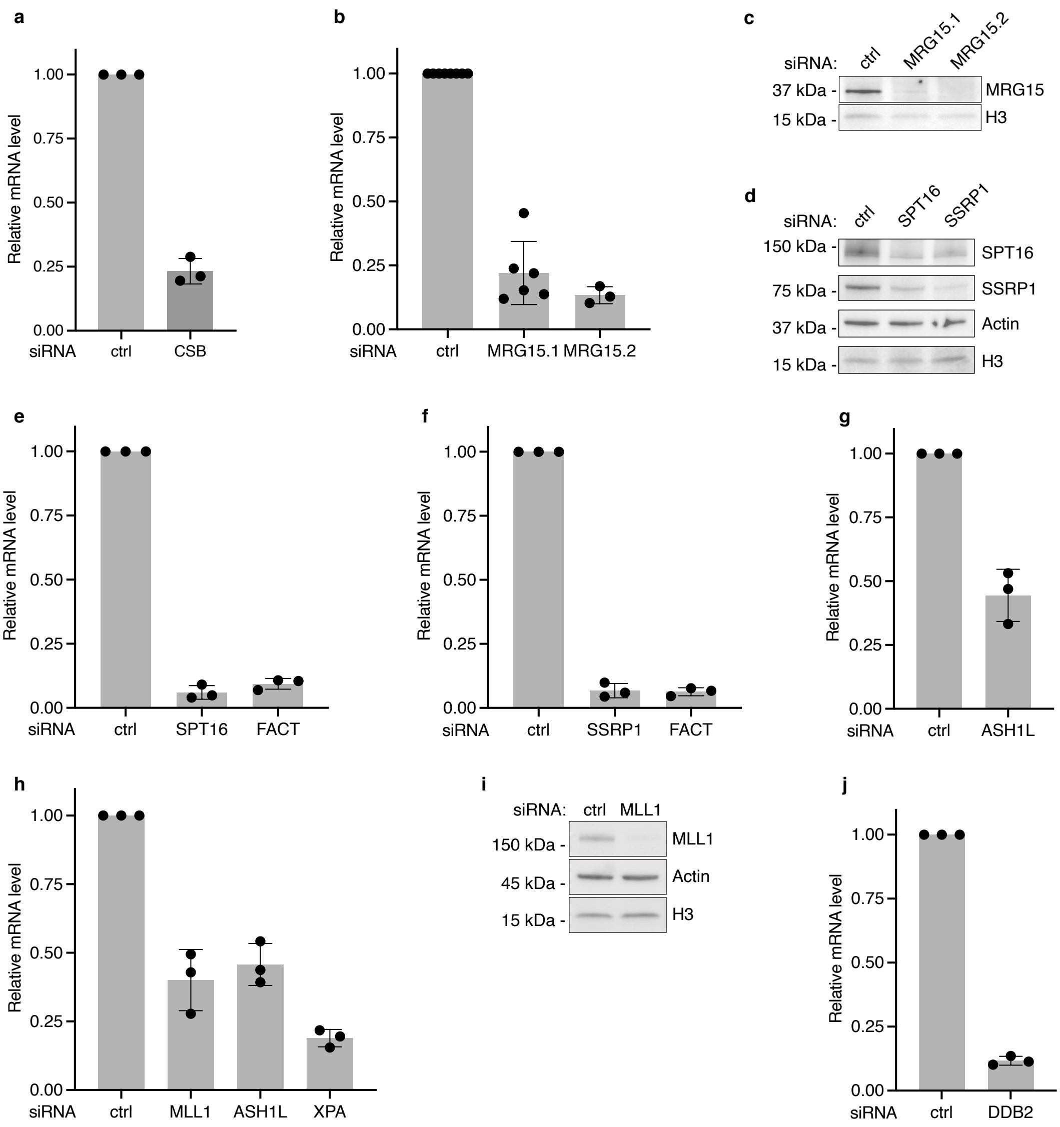

Supplementary Fig. 3. Efficiency of factor depletions by siRNA transfections. The efficiency of each siRNA treatment was demonstrated by PCR amplification of the target mRNA or immunoblotting of the respective translated proteins. **a** Depletion of the mRNA coding for CSB. Mean values ± SD (n = 3 independent experiments). **b** Depletion of the mRNA coding for MRG15 by two different siRNA sequences. Mean values ± SD (n = 3 or 5 independent experiments, as indicated). **c** Depletion of MRG15 protein by two different siRNA sequences. **d** Depletion of SPT16 and SSRP1 proteins. The siRNA-mediated depletion of one FACT subunit also results in a reduction of the other subunit. **e** and **f** Depletion of the mRNA coding for SSRP1 and SPT16 by siRNA targeting SSRP1 or SPT16 alone, or a combination of the two siRNA sequences (denoted as siRNA against FACT). Mean values ± SD (n = 3 independent experiments). **g** Depletion of the mRNA coding for ASH1L. Mean values ± SD (n = 3 independent experiments). **h** Depletion of the mRNA coding for MLL1, ASH1L or XPA. Mean values ± SD (n = 3 independent experiments). **i** Depletion of MLL1 protein. **j** Depletion of the mRNA coding for DDB2. Panels **a**, **b**, **e**, **f**, **g**, **h** and **j** show mean values ± SD (n = 3 independent experiments). For the experiments of **c**, **d** and **i**, two independent replicates were conducted obtaining similar results.

a

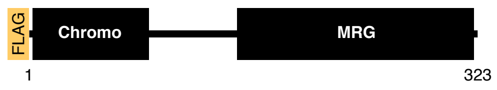

b

Protein name	Total unique peptide count	
	WT	Domain mutant
DDB1	36	15
Cul4A	6	0
DDB2	3	1
DCAF1	12	2
DCAF8	6	2

c

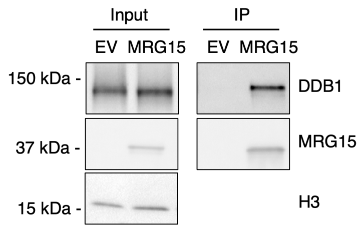

Supplementary Fig. 4. MRG15 recruitment by the DDB1-DDB2 complex. The Flag tag-mediated immunoprecipitation of MRG15 revealed a previously unknown interaction with DDB1. **a** Domain structure of MRG15. **b** Mass spectrometric analysis of immunoprecipitates: number of peptides identified for the listed proteins using wildtype (WT) MRG15 or a mutant (Tyr235Ala substitution in the MRG domain). Mean values of two independent experiments. **c** Immunoblot analysis of immunoprecipitates (IP) confirming the interaction of MRG15 with DDB1. EV, empty vector. Input: 10% of total cell lysate. Two independent replicates were conducted obtaining similar results.

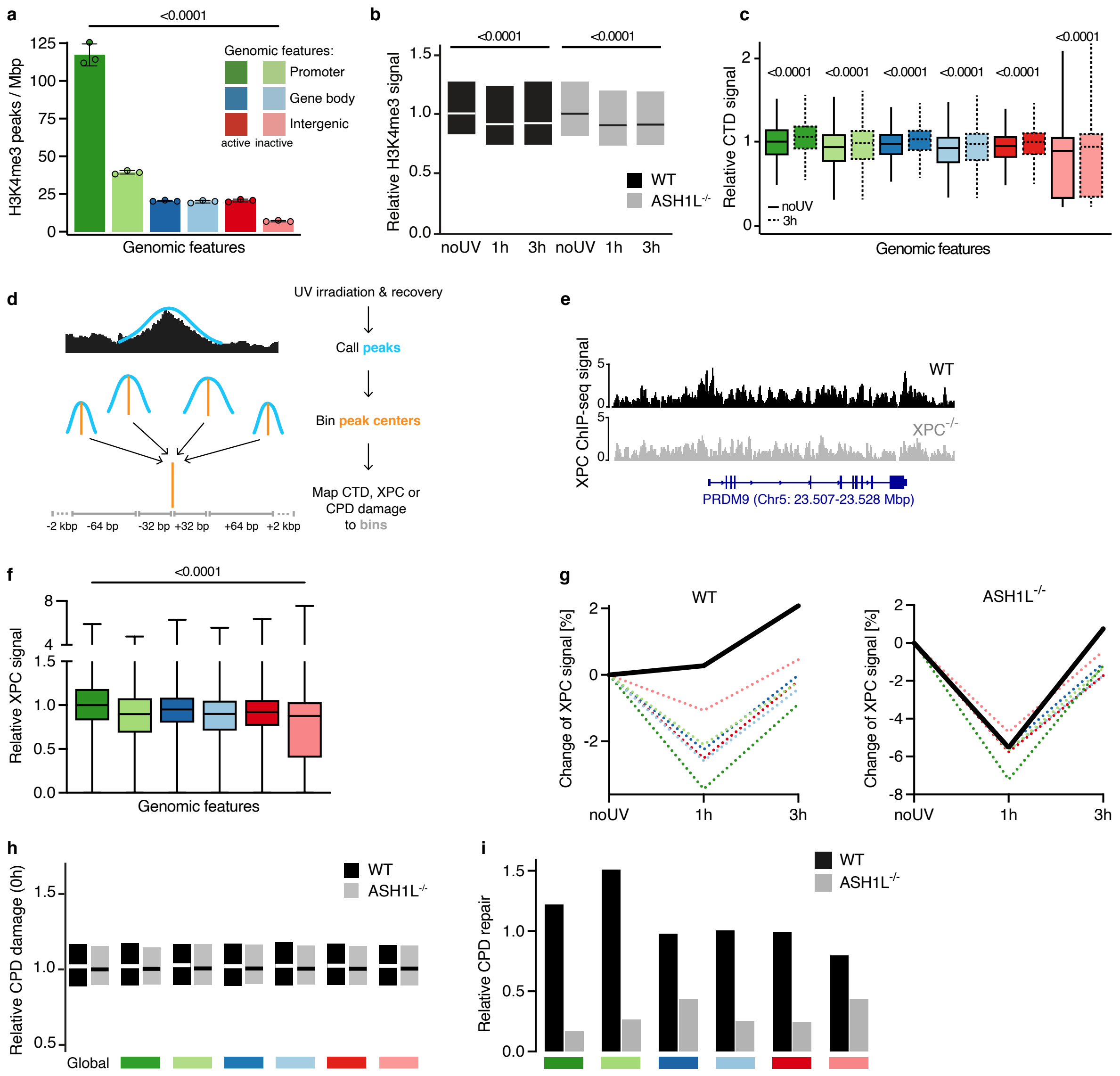

Supplementary Fig. 5. ChIP-seq and CPD-damage seq. a The density of unfiltered H3K4me3 peaks is higher in active promoters than at other genomic features in WT cells (see inset for the color code). Mean values of three independent experiments ± SD; one-way ANOVA. **b** Overall UV-induced changes of the H3K4me3 signal in WT and ASH1L$^{-/-}$ cells. The UV irradiation dose was 20 J·m^{-2}. Boxplots show medians (normalized to the H3K4me3 signal in unchallenged WT cells), first and third quartiles (n = 3 independent experiments). Two-tailed paired Wilcoxon rank sum test. **c** Increase of the overall CTD signal in WT cells in response to UV irradiation (n = 2 independent experiments). Boxplots show medians (normalized to the CTD signal in unirradiated cells), first and third quartiles. Whiskers extend from minimum to maximum values (Mann Whitney *U* test, two-tailed). **d** Scheme illustrating how sequencing reads were combined to test for positional correlations. The centers of methylation peaks were compiled into a DNA segment ("bin") spanning 64 bp. These center bins were expanded out by increments of 64 bp, thus covering up to ± 2 kilobase pairs away from the peak centers. Sequencing reads derived from CTD-ChIP-seq, XPC-ChIP-seq or HS-damage-seq were mapped to each bin of this region of ± 2 kilobases. Similarly, CPDs and CPD excision rates (during the first 3 h after the UV pulse) were mapped around the center of histone methylation peaks. Lines were finally plotted using the locally weighted scatterplot smoothing method with a default span of 0.75; shaded areas around each line represent 95%

confidence intervals. **e** ChIP-seq tracks obtained from unirradiated WT and XPC$^{-/-}$ cells using anti-XPC antibodies. The profiles span 21 kilobase pairs of chromosome 5. **f** Baseline XPC occupancy obtained by four independent ChIP-seq analyses of unchallenged WT cells (same color code as in **a**). Boxplots show medians (normalized to the XPC signal in active promoters), first and third quartiles. Whiskers extend from minimum to maximum values (one-way ANOVA). **g** Percentage changes of XPC occupancy in different genomic features of WT (panel on the left) and ASH1L$^{-/-}$ cells (panel on the right), 1 and 3 h after UV irradiation (mean values of three independent experiments). Thick black line, changes of XPC occupancy at sites of novel ASH1L-deposited H3K4me3 peaks; dotted lines, changes of XPC occupancy in the different genomic features (same color code as in **a**). **h** Baseline CPD frequency after UV irradiation of WT and ASH1L$^{-/-}$ cells, at the global-genome level and in the different genomic features (same color code as in **a**; n = 1 HS damage-seq experiment). Boxplots show medians (normalized to the global CPD frequency in WT cells), first and third quartiles. **i** Normalized CPD excision over 3 h after UV irradiation in the indicated genomic features (n = 1 HS damage-seq experiment). Black, WT cells; grey, ASH1L$^{-/-}$ cells. The data of Fig. 3f were corrected for the varying proportion of DNA in each genomic feature.

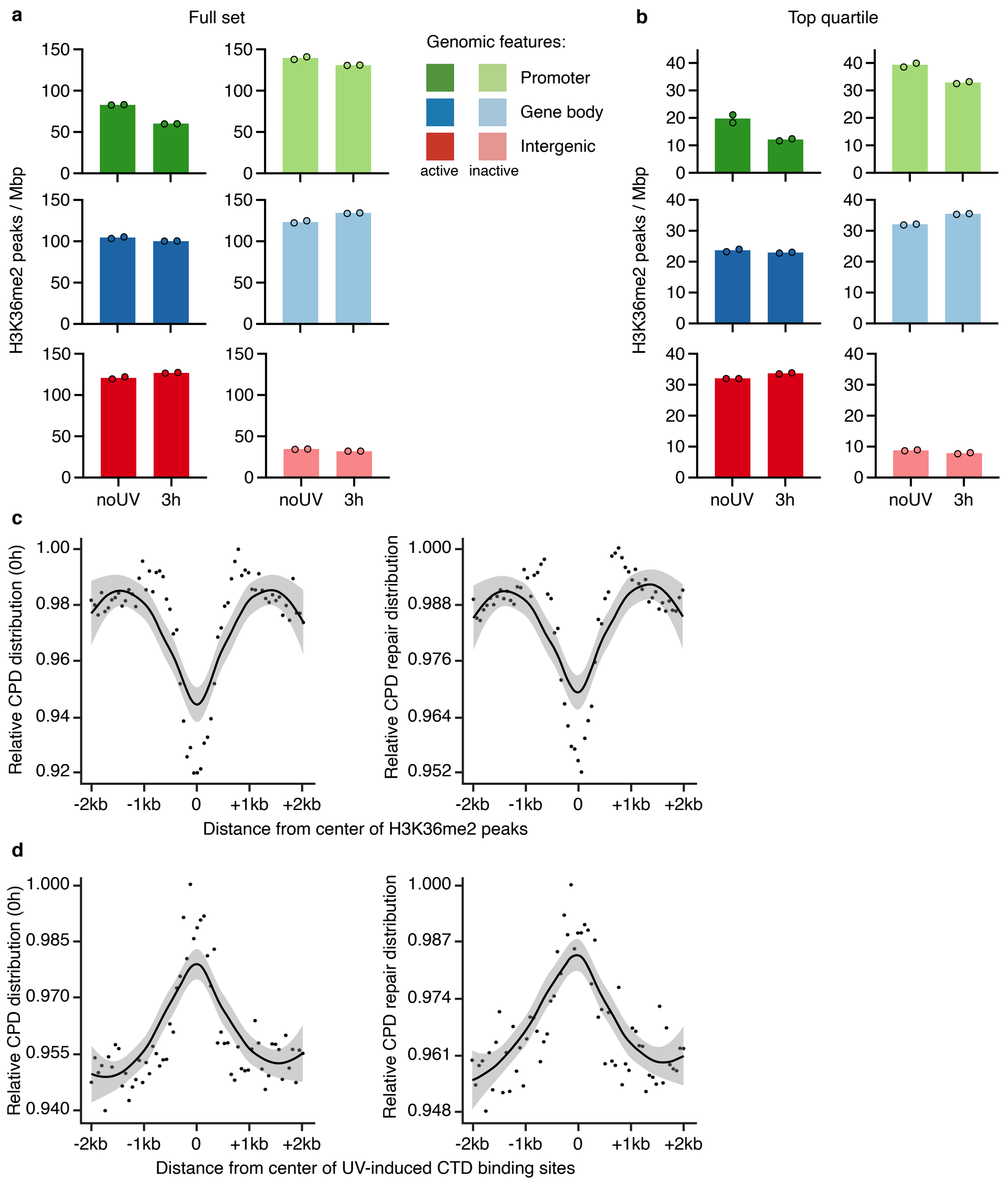

Supplementary Fig. 6. H3K36me2 marks occur away from CPD sites and CTD occupancy. a Density of unfiltered H3K36me2 peaks obtained by ChIP-seq analysis on WT cells before or after a 3-h recovery post UV (20 J·m^{-2}). Unlike H3K4me3, the H3K36me2 peak density is lower in active promoters than in most other genomic features, and not substantially increased upon the UV challenge (mean values of two independent experiments). **b** The H3K36me2 tracks were filtered for peak height with the top quartile as the threshold (mean values of two independent experiments). **c** The ~100,000 genomic sites harboring *de novo* H3K36me2 peaks deposited during 3 h post UV, and their flanking sequences, were subdivided into 64-bp bins. The mean initial abundance of CPDs is indicated for each of these bins. Panel on the left: genome-wide positional correlation demonstrating that H3K36me2 is added preferentially away from CPD sites. Panel on the right: low CPD excision rates in the center of novel H3K36m2 peaks deposited after UV irradiation. The dip in the center of the H3K36me2 peaks is indicative of poor CPD repair. These plots were generated as outlined in Supplementary Fig. 5d (shaded areas around each line represent 95%

confidence intervals). **d** Distribution of CTD occupancy relative to initial CPD formation and CPD excision. The peaks of the CTD of ASH1L detected 3 h after UV were filtered with the 75th percentile as the threshold. Shown are CPD densities around the center of these CTD peaks (panel on the left) and CPD excision rates during a 3-h recovery, again relative to the center of CTD peaks (panel on the right). Shaded areas around each line represent 95% confidence intervals.

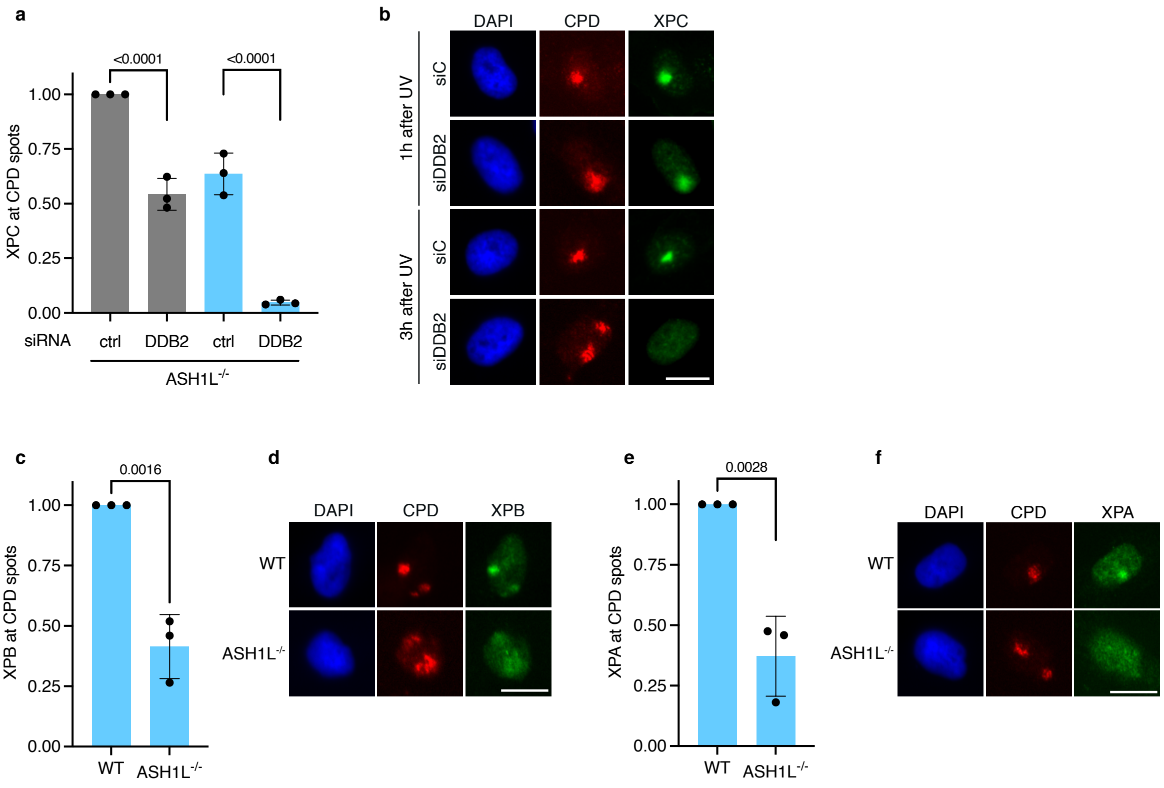

Supplementary Fig. 7. DDB2- and ASH1L-dependent factor recruitment. DDB2 and ASH1L are indispensable for the efficient recruitment of GG-NER factors to UV lesion sites. **a** Upon siRNA-mediated depletion of DDB2, the recruitment of XPC to UV lesion spots is diminished 1 h (grey bars) and completely abrogated 3 h (blue bars) after UV irradiation. Quantification of three independent experiments relative to local XPC levels at the 1-h time point in cells transfected with control siRNA. Mean values ± SD (one-way ANOVA). **b** Images showing that the recruitment of XPC is dependent on DDB2, particularly at the 3-h time point after UV irradiation, when most pyrimidine-pyrimidone (6-4) photoproducts have been removed. **c** ASH1L$^{-/-}$ cells are impaired in the recruitment of XPB to UV lesion spots 3 h post UV. Quantification of three independent experiments relative to local XPB levels in WT cells. Mean values ± SD (two-tailed t-test). **d** Images showing that, at 3 h post UV, ASH1L-deficient cells are impaired in the XPB recruitment to UV lesions. **e** ASH1L$^{-/-}$ cells are impaired in the recruitment of XPA to UV lesion spots 3 h post UV. Quantification of three independent experiments relative to local XPA levels in WT cells. Mean values ± SD (two-tailed t-test). **f** Images showing that, at 3 h post UV, ASH1L-deficient cells are impaired in the XPA recruitment to UV lesions. Scale bars in **b**, **d** and **f** correspond to 15 µm.

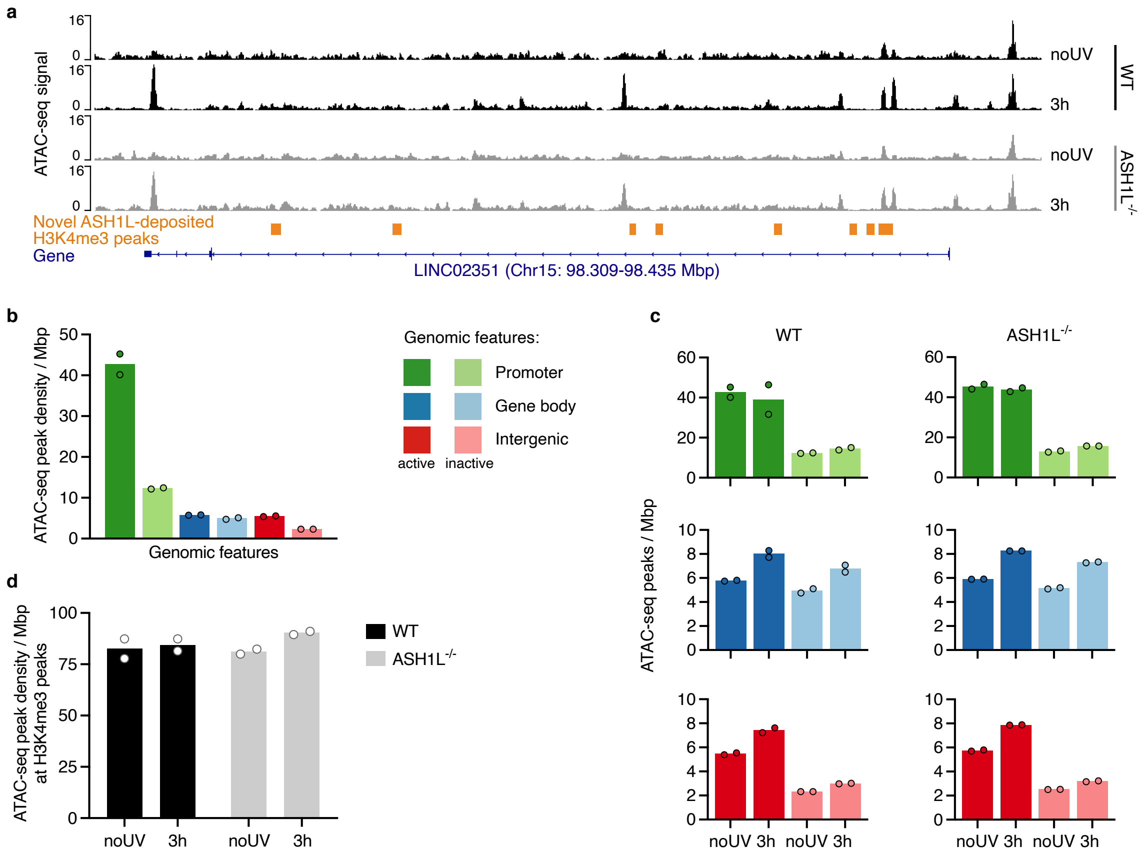

Supplementary Fig. 8. ASH1L does not contribute to the UV-induced increase in chromatin accessibility. a Representative tracks showing UV-dependent increases in chromatin accessibility (in WT and ASH1L$^{-/-}$ cells), proxied by ATAC-seq signals, at a genic region of chromosome 15 spanning 126 kilobase pairs. The UV dose was 20 J·m^{-2} in all experiments. **b** Under unchallenged conditions, the strongest (top quartile) accessibility peaks map predominantly to active gene promoters (data normalized for the varying proportion of DNA in each genomic feature). Heterochromatic intergenic regions were, as expected, the least accessible. Mean values of two independent experiments. **c** The ATAC-seq peak density significantly increases upon UV irradiation at genes and intergenic regions, but ASH1L does not contribute to these changes in accessibility. Barplots were derived using the top quartile of ATAC-seq peaks (mean values of two independent experiments). **d** The ATAC-seq analysis demonstrates that DNA accessibility does not change at ASH1L-deposited H3K4me3 peaks upon UV radiation (top quartile of ATAC-seq peaks, mean values of two independent experiments).

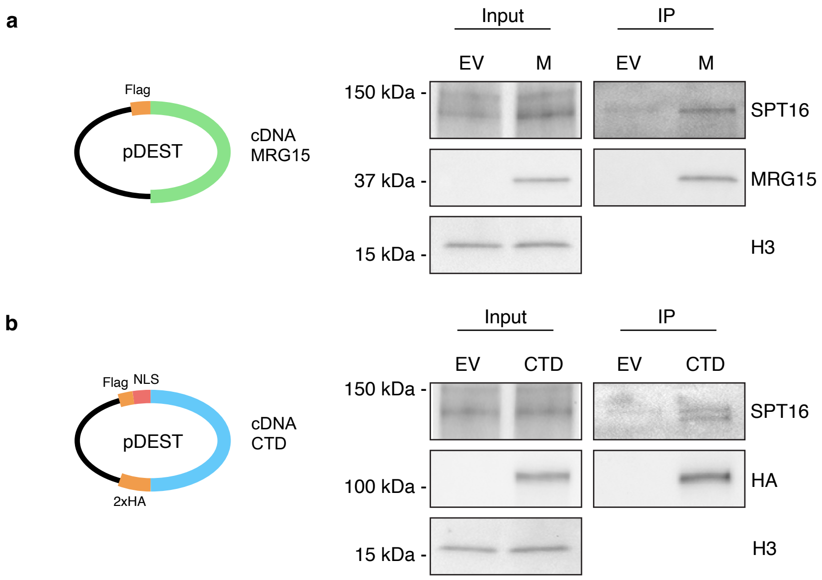

Supplementary Fig. 9. Co-existence of MRG15-ASH1L and FACT in protein complexes. **a** The Flag tag-mediated immunoprecipitation of MRG15 (expressed in HEK293T cells from the pDEST vector) confirmed an interaction with the SPT16 subunit of FACT. Input: 10% of total cell lysate, solubilized after benzonase treatment. EV, transfection with empty vector; M, transfection with MRG15 vector. **b** The Flag tag-mediated immunoprecipitation of CTD (expressed in HEK293T cells from the pDEST vector) confirmed an interaction with the SPT16 subunit of FACT. Input: 10% of total cell lysate, solubilized after benzonase treatment. EV, transfection with empty vector; transfection with CTD vector. For the experiments of **a**, **b**, two independent replicates were conducted obtaining similar results.

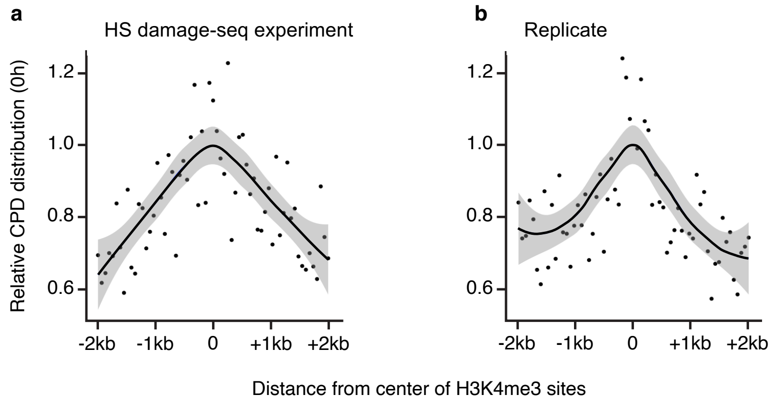

Supplementary Fig. 10. Replicability of CPD profiles determined by HS damage-seq. U2OS cells were subjected to HS damage-seq in two separate experiments. The ~55,000 sites of novel ASH1L-dependent H3K4me3 peak formation across the genome were used as a landmark to compare damage distributions immediately after UV irradiation (20 $J{\cdot}m^{-2}$). To that end, the CPDs were binned around the center of histone methylation sites as outlined in Supplementary Fig. 5d (shaded areas around each line represent 95% confidence intervals). **a** CPD distribution in the damage-seq data used to generate Figs. 3 and 4, based on the higher read count capturing 50 million CPDs. **b** CPD distribution in the replicate experiment, having a lower read count of 32 million CPDs. The similarity of the two CPD distribution profiles throughout the genome supports the robustness of the HS damage-seq data.

Supplementary Table 1. Common interactors of XPC, CTD and MRG15 detected by mass spectrometric analysis of immunoprecipitates. The table lists the peptide counts for each interactor following immunoprecipitation with the indicated baits.

**Protein**	**XPC**	**CTD**	**MRG15**
PARP1	33	3	5
PP1A	16	1	8
HSPB1	6	4	13
H1X	6	1	2
CBX3	7	3	3
SAP18	5	2	8
MAGB2	3	2	7
SPT16	5	2	3
DNJC9	1	1	3
ACL6A	1	2	2
RL24	2	2	3
RL17	10	4	6
FXR1	13	2	13
RL18A	8	2	6
EF2	3	1	16
RL35	6	2	7
RL21	7	3	10
RL31	6	1	5
RL23	6	1	5
RS24	5	1	4
RS26	2	2	1
YTDC1	6	1	7
RL22	4	1	4
RS23	4	3	4
RL30	6	2	3
SRP68	2	1	9
RS30	3	3	2
PPIL1	2	3	3

Supplementary Table 2. List of antibodies, their supplier and uses.

Antibody target	Species	Supplier, catalog number	Uses and dilutions
**Primary antibodies:**			
Actin	mouse	Millipore, MAB1501	Western blot (WB) 1:5000
ASH1L	mouse	Santa Cruz, sc-98301	WB 1:500
ASH1L	rabbit	Novusbio, NB100-93290	WB 1:500
CPD	mouse	Cosmo, NMDND001	Immunofluorescence (IF) 1:1000
DDB2	mouse	Abcam, ab51017	WB 1:500
Flag M2	mouse	Sigma, F1804	Chromatin immunoprecipi-tation (ChIP), WB 1:1000
H3	goat	Santa Cruz, sc-8654	WB 1:500
H3K4me3	rabbit	Abcam, ab8580	Chromatin immunoprecipi-tation (ChIP), WB 1:1000
H3K36me2	rabbit	Abcam, ab9049	Chromatin immunoprecipi-tation (ChIP), WB 1:1000
HA	mouse	Abcam, ab18181	WB 1:1000
MRG15	rabbit	Bioworld, BS71896	IP, WB 1:500
SPT16	rabbit	Abcam, ab204343	IF 1:200
SPT16	mouse	Santa Cruz, sc-377028	WB 1:500
SSRP1	mouse	Santa Cruz, sc-74536	WB 1:500
MLL1	rabbit	Cell Signaling, 14197S	WB 1:1000
XPA	rabbit	Santa Cruz, sc-853	WB 1:500, IF 1:50
XPB	rabbit	Santa Cruz, sc-293	IF 1:100
XPC	rabbit	Sigma, X1129	IF 1:100
XPC	mouse	Santa Cruz, sc-74411	WB 1:500
XPC	rabbit	Invitrogen, PA5-97019	ChIP, WB 1:1000
XPD	rabbit	GeneTex, GTX108948	WB 1:1000, IF 1:100
**Secondary antibodies:**			
Anti-mouse IgG-DyLight 594	goat	Invitrogen, 35510	IF 1:400
Anti-rabbit IgG-Alexa Fluor 488	goat	Invitrogen, A11034	IF 1:400
Anti-goat IgG-peroxidase	rabbit	Sigma, A5420	WB 1:5000
Anti-mouse IgG-peroxidase	goat	Sigma, A2304	WB 1:5000
Anti-rabbit IgG-peroxidase	goat	Sigma, A0545	WB 1:5000
F(ab')2 anti-mouse IgG-biotin	goat	Thermofisher, A24516	Enzyme-linked immunoassay 1:2000

Supplementary Table 3. List of siRNA sequences used for protein depletions by RNA interference.

siRNA	Sequence 5'-3'	Supplier, catalog number
siCtrl (control)	AAUUCUCCGAACGUGUCACGUTT	Microsynth
siASH1L	CAGGCUGUCCUAUCAAUGCAATT	Microsynth
siCSB	GAAGCAAGGUUGUAAUAAATT	Microsynth
siMRG15.1	AAGCAGAAACAGCGAGAACTT	Microsynth
siMRG15.2	AAGCCAAUCAGGAGCAGUATT	Microsynth
siSPT16	SMARTpool	Dharmacon, M-009517-00-0005
siSSRP1	SMARTpool	Dharmacon, M-011783-01-0005
siMLL1	SMARTpool	Dharmacon, M-009914-01-0005
siXPA	GCUACUGGAGGCAUGGCUA	Microsynth
siDDB2	AGGGAUCAAGCAGUUAUUUGA	Microsynth

Supplementary Table 4. List of oligonucleotides, their supplier and uses.

Oligonucleotide	Sequence 5'-3'	Supplier	Uses
CTD forward	GGGGACAAGTTTGTACAAAAAAGCAGGCTTCACCATGGGAAAGTATCTAAGACAAAAGAGAATTGAC	Microsynth	GATEWAY cloning
CTD reverse	GGGGACCACTTTGTACAAGAAAGCTGGGTCCTACTTTCGAAAGCTGTTTTCTGG		
$CTD_{inactive}$ forward	CTTTCATTCCGCCAATGTGGAAAAACAGCAACTTTG	Microsynth	Mutagenesis
$CTD_{inactive}$ reverse	GTTTTTCCACATTGGCGGAATGAAAGTTATAATCATAAGTG		
MRG15 MRG domain mutant forward	CCAGCTACTCGCCAAATTTGAGAGACCAC	Microsynth	
MRG15 MRG domain mutant forward	CTCTCAAATTTGGCGAGTAGCTGGGTACC		
ASH1L forward	TATTGAAGTGGCTCGGGCAG	Microsynth	RT-PCR primers
ASH1L reverse	CACACAGTCACCCTGACGAA		
MRG15 forward	ATGGTGGCAGTACCAGTGAGAC	Microsynth	
MRG15 reverse	GCCACGGTTTTAGCTCTTCAGG		
SPT16 forward	GTGGAAAAGGCCATTGAAGA	Microsynth	
SPT16 reverse	GTGATAGCCCCAAAGTGCAT		
SSRP1 forward	AGGCAAGAATGAGGTGACA	Microsynth	
SSRP1 reverse	TACATCCGCCTTTGACAACA		
CSB forward	CGGATCCAGATCTCGAGCTCATGTACCCATACG	Microsynth	
CSB reverse	CGTATGGGTACATGAGCTCGAGATCTGAGTCCG		
MLL1 forward	GTGCTTTGTGGTCAGCGGAAGT	Microsynth	
MLL1 reverse	TGTGAGACAGCAACCCACGGTG		
DDB2 forward	AACCCAGAAGACCTCCGAGA	Microsynth	
DDB2 reverse	ACATCTTCTGCTAGGACCGGA		
XPA forward	GGCGAGTATCGAGCGGAAG	Microsynth	
XPA reverse	TGAAGCCTCCTCCTGTGTCA		
ASH1L exon 2 forward	TCCTTGGCAATAACATACGCT	Microsynth	Primers for sequencing
ASH1L exon 2 reverse	TGGCAGGTCGACATACATAGT		
ASH1L exon 11/12 forward	TTGCAGCCCGACTTGGAAAT	Microsynth	
ASH1L exon 11/12 reverse	CCTGAGCTGCTTTGTGTAAGC		
XPC exon 1 forward	CTCGCGAAGTGGAATTTGCC	Microsynth	
XPC exon 1 reverse	TCGCTCTCACCCTCCTCC		
XPC exon 2 forward	CAGGGGATGGTGAACCAGTG	Microsynth	
XPC exon 2 reverse	ATCCAATCTTCCATGGACCCC		
XPA exon 1 forward	AGCTAGGTCCTCGGAGTGG	Microsynth	

XPA exon 1 reverse	AGAATCTGCACACATACGCCA		
XPF exon 1 forward	GAGCTTCCATGGAGTCAGGG	Microsynth	
XPF exon 1 reverse	TCCTCCTAGCGACCCCTTAC		
XPF exon 2 forward	CCTGGCTTTGGTGGGAAGTA	Microsynth	
XPF exon 2 reverse	AGGGAGCTGAGTCCTTCAAGA		
AD1T	phos-GATCGGAAGAGCACACGTCTGAACTCCAGTCA-SpC3	Eurogentec	HS damage-seq
AD1B	NNNNNGACTGGTTCCAATTGAAAGTGCTCTTCCGATC*T	Eurogentec	
AD2T	phos-AGATCGGAAGAGCGTCGTGTAGGGAAAGAGTGT-SpC3	Eurogentec	
AD2B	ACACTCTTTCCCTACACGACGCTCTTCCGATCTNNNNN-SpC3	Eurogentec	
O3P	biotin-GACTGGAGTTCAGACGTGTGCTCTTCCGATCT	Eurogentec	
SH	biotin-NNGACTGGTTCCAATTGAAAGTGCTCTTCCG-SpC3	Eurogentec	
N701 Nextera adapter	CAAGCAGAAGACGGCATACGAGATTCGCCTTAGTCTCGTGGGCTCGG	Microsynth	ATAC-seq amplifica-tion of tagmented libraries
N702 Nextera adapter	CAAGCAGAAGACGGCATACGAGATCTAGTACGGTCTCGTGGGCTCGG	Microsynth	
N703 Nextera adapter	CAAGCAGAAGACGGCATACGAGATTTCTGCCTGTCTCGTGGGCTCGG	Microsynth	
N704 Nextera adapter	CAAGCAGAAGACGGCATACGAGATGCTCAGGAGTCTCGTGGGCTCGG	Microsynth	
N705 Nextera adapter	CAAGCAGAAGACGGCATACGAGATAGGAGTCCGTCTCGTGGGCTCGG	Microsynth	
N706 Nextera adapter	CAAGCAGAAGACGGCATACGAGATCATGCCTAGTCTCGTGGGCTCGG	Microsynth	
N501 Nextera adapter	AATGATACGGCGACCACCGAGATCTACACTAGATCGCTCGTCGGCAGCGTC	Microsynth	
N502 Nextera adapter	AATGATACGGCGACCACCGAGATCTACACCTCTCTATTCGTCGGCAGCGT	Microsynth	
N503 Nextera adapter	AATGATACGGCGACCACCGAGATCTACACTATCCTCTTCGTCGGCAGCGTC	Microsynth	
N504 Nextera adapter	AATGATACGGCGACCACCGAGATCTACACAGAGTAGATCGTCGGCAGCGTC	Microsynth	

*phosphorothioate bond

Supplementary Table 5. Summary of statistical analyses.

Fig.	Test	F value	Degree of freedom	t value	P values
**Main figures**					
1c	One-way ANOVA	38.06	8		0.0042
1g	One-way ANOVA	406.3	17		See individual values in Fig. 1g
1h	Two-tailed t-test		4	5.182	0.0066
2e	One-way ANOVA	17.94	27		See individual values in Fig. 2e
2h	One-way ANOVA	70.4	12		< 0.0001
2k	One-way ANOVA	66.24	11		See individual values in Fig. 2k
3a	One-way ANOVA	17528	17		< 0.0001
3b	One-way ANOVA (TSS WT)	2493	17		See individual values in Fig. 3b
	One-way ANOVA (TSS ASH1L$^{-/-}$)	707.3	17		
	One-way ANOVA (GB WT)	58.71	17		
	One-way ANOVA (GB ASH1L$^{-/-}$)	1.826	17		
	One-way ANOVA (IG WT)	289	17		
	One-way ANOVA (IG ASH1L$^{-/-}$)	12.34	17		
3f	Two-tailed paired Wilcoxon rank sum test				See individual values in Fig. 3f
3g	Two-tailed unpaired Wilcoxon rank sum test				See individual values in Fig. 3g
5a	One-way ANOVA	10.84	17		See individual values in Fig. 5a
5c	Two-tailed t-test		4	7.765	0.0015
5e	One-way ANOVA	11.39	14		See individual values in Fig. 5e
5g	Two-tailed t-test		4	28.21	< 0.0001
5i	Two-tailed t-test		4	5.758	0.0045
5k	One-way ANOVA	3.954	14		0.046
6b	One-way ANOVA	98.31	14		< 0.0001
6d	Two-tailed t-test		8	8.454	< 0.0001
6f	Two-tailed t-test		8	39.14	< 0.0001
6g	One-way ANOVA	102	11		< 0.0001
6i	One-way ANOVA	68.14	17		See individual values in Fig. 6i
6j	One-way ANOVA	14.4	8		See individual values in Fig. 6j
**Supplementary figures**					
2e	Two-way ANOVA	24.71	3		See individual values in Suppl. Fig. 2e
5a	One-way ANOVA	521.1	17		< 0.0001

5b	Two-tailed paired Wilcoxon rank sum test				< 0.0001 for all comparisons
5c	Two-tailed Mann Whitney *U* test				< 0.0001 for all comparisons
5f	One-way ANOVA	5506	868291		< 0.0001 for all comparisons
7a	One-way ANOVA	126.8	11		See individual values in Suppl. Fig. 7a
7c	Two-tailed t-test		4	7.63	0.0016
7e	Two-tailed t-test		4	6.568	0.0028

Supplementary Table 6. List of data analysis tools.

Tool	Pipeline	Reference
TrimGalore v.0.6.5	ChIP-seq	6
macs2 v.2.2.7.1	ChIP-seq, ATAC-seq	7
idr v.2.0.4.2	ChIP-seq, ATAC-seq	8
bbmap v.38.90	HS DNA damage-seq	9
bwa mem v.07.17-r1188	ChIP-seq, HS DNA damage-seq	10
samtools v.1.7	All genomics data pipelines	11
bedtools v.2.29.2	All genomics data pipelines	12
Picard v.2.23.8	ChIP-seq, ATAC-seq	http://broadinstitute.github.io/picard
R v.4.0.3, v.4.1.0	All genomics data pipelines	13
Rcpp v.1.0.8.3	ChIP-seq, ATAC-seq	14
edgeR v.3.34.1	HS DNA damage-seq	15
DESeq2 v.1.32.0	ChIP-seq	16
ggpubr v.0.4.0	All genomics data pipelines	17
ggplot2 v.3.3.5	All genomics data pipelines	18
dplyr v.1.0.8	All genomics data pipelines	19
tidyr v.1.2.0	All genomics data pipelines	20
rstatix v.0.7.0	All genomics data pipelines	21
cutadapt v.1.9.1	ATAC-seq	6
bowtie2 v.2.4.5	ATAC-seq	22
MaxQuant v.2.0.1.0	Mass spectometry	5

Andromeda search engine	Mass spectometry	23
Image Studio v.5.2.5	Clonogenic survival assay	None
ImageJ v.2.8.0	Unscheduled DNA synthesis assay	24
FlowJo v10.9	Flow cytometry	26

Supplementary methods
HS damage-seq
Oligonucleotides used for library preparation are listed in Supplementary Table 4. AD1 and AD2 adaptors (40 μM) were prepared by mixing equal volumes (20 μl) of 100 μM AD1T/AD2T and AD1B/AD2B with 10 μl 5x annealing buffer (50 mM Tris-HCl, pH 8.0, 250 mM NaCl, 5 mM EDTA), heating to 98°C, then allowing to slowly cool to 25°C. The anti-CPD antibody was reconstituted with 100 μl of distilled water, then mixed with 100 μl glycerol and stored at -20°C.

Genomic DNA (1.5 μg) extracted from U2OS cells was sheared using a Q800 sonicator (Qsonica) to produce fragments of on average 400-bp length, using the following program: 20% amplitude for 3 min, 2 sec on/5 sec off. The fragmented DNA was subjected to size-selective purification to remove fragments < 200 bp with 1x volume of AMPure XP DNA purification beads (Beckman Coulter). Next, the size-selected DNA (1 μg) was used for end preparation and AD1 (40 μM) ligation according to the instructions of NEB Next Ultra II DNA Library Prep Kit for Illumina (New England Biolabs). The ligation mixture was kept 16 h at 4°C for adequate ligation. The ligation product was purified with 0.7x volume (65 μl) of AMPure XP and eluted with 12 μ 0.1x TE (50 mM Tris-HCl, 5 mM EDTA) buffer. Eluted DNA was denatured by mixing with 5 μl of 8 M urea, heated at 98°C for 2 min, and immediately placed on ice. The denatured DNA was mixed with 0.5 μl of salmon sperm DNA (10 mg/mL, Thermo Fisher Scientific, AM9680), 2.5 μl of pre-cooled 8x IP buffer [160 mM Tris-HCl, pH 8.0, 16 mM EDTA, 1.2 M NaCl, 8% (vol/vol) Triton X-100, 4% (wt/vol) sodium deoxycholate] and antibody-coated beads, which were prepared as follows. Beads were coated with antibodies by first mixing 1.25 μl of protein G Dynabeads (Thermo Fisher Scientific, 10003D,) and 1.25 μl anti-rabbit Dynabeads (Thermo Fisher Scientific, 11203D). They were washed twice using 100 μl pre-cooled 1x IP buffer, followed by addition of 5 μl 1x IP buffer, 0.25 μl salmon sperm DNA, 0.5 μl rabbit anti-mouse IgG (Abcam, ab6709) and 0.5 μl of reconstituted anti-CPD antibody. The beads were suspended gently by pipetting to avoid inducing bubbles, then rotated at 4°C overnight on a tube revolver (Thermo Fisher Scientific, 88881001) in the oscillation mode. The antibody-coated beads were washed using 100 μl pre-cooled 1x IP buffer and mixed with the DNA solution described above. The mixture was suspended and rotated for immunoprecipitation at 4°C overnight on the tube revolver in oscillation mode. The beads obtained from immunoprecipitation were washed sequentially with the following buffers: Wash buffer U [20 mM Tris-HCl, pH 8.0, 2 mM EDTA, 1% (vol/vol) Triton X-100, 2 M Urea], Wash buffer I [20 mM Tris-HCl, pH 8.0, 2 mM EDTA, 150 mM NaCl, 1% (vol/vol) Triton X-100, 0.1% (wt/vol) SDS], Wash buffer II [20 mM Tris-HCl, pH 8.0, 2 mM EDTA, 500 mM NaCl, 1% (vol/vol) Triton X-100, 0.1% (wt/vol) SDS], Wash buffer III [10 mM Tris-HCl, pH 8.0, 1 mM EDTA, 150 mM LiCl, 1% (vol/vol) NP-40, 1% (wt/vol) sodium deoxycholate], Wash buffer IV [100 mM Tris-HCl, pH 8.0, 1 mM EDTA, 500 mM LiCl, 1% (vol/vol) NP-40, 1% (wt/vol) sodium deoxycholate] and 1x TE buffer. The washing step was performed by adding 200 μl wash buffer, resuspending by vortexing, and rotating on the tube revolver for 2 min. The washed beads were eluted twice with 50 μl pre-warmed elution buffer [10 mM Tris-Cl pH 8.0, 1 mM EDTA, 1% (wt/vol) SDS] at 65°C and 120 g for 5 min. The DNA in a combined elution solution was purified by phenol-chloroform extraction and precipitated by adding 10 μl of 3 M sodium acetate, 1 μl GlycoBlue (Thermo Fisher Scientific, AM9515) as co-precipitant and 250 μl of ethanol. The pellet was air dried and resuspended in 6 μl 0.1x TE buffer.

The purified DNA was mixed with 1.5 μl of O3P primer (20 μM) and an equal volume of NEBNext Ultra II Q5 Master Mix (New England Biolabs), then incubated under the following conditions in thermocycler T100 (Bio-Rad Laboratories) for polymerase extension: 50 sec at 98°C, 5 min at 65°C and on hold at 37°C. To digest the excessive amount of primer, 1.5 μL exonuclease I (New England Biolabs, M0293) were added to the extension mixture,

followed by incubation at 37°C for 15 min. The mixture was purified with a 0.9x volume of AMPure XP (37 µl, 25 µl MilliQ) and eluted with 20 µl 0.1x TE buffer. The eluate was mixed with 2 µl of SH primer (10 µM), 25 µl 1x B&W buffer [5 mM Tris-HCl, pH 8.0, 0.5 mM EDTA, 1 M NaCl, 0.1% (vol/vol) Tween 20] and subjected to a slow annealing process using the thermocycler under the following conditions: 2 min at 98°C, then cooling at 1 min/°C from 97°C to 76°C, 5 min/°C from 75°C to 55°C, 1 min/°C from 54°C to 25°C, and on hold at 4°C. The annealing product was stored at -20 °C for later processing. Next, 10 µl Dynabeads MyOne Streptavidin C1 (Thermo Fisher Scientific, 65001) were washed twice with 1x B&W buffer, resuspended with 5 µl 5x binding buffer [50 mM Tris-HCl, pH 8.0, 5 mM EDTA, 2.5 M NaCl, 0.1% (vol/vol) Tween 20, 0.1% (wt/vol) CA-630, 25 mM $MgCl_2$)] and added to the annealing product. The mixture was rotated 1 h at 4°C on the tube revolver in oscillation mode. The supernatant was transferred to a new 1.5-ml tube, the beads were washed with 50 µL 1x B&W buffer, and the supernatants were pooled. The DNA in pooled supernatants was purified by ethanol precipitation by adding 1 µl GlycoBlue and 250 µl of ethanol. The air-dried pellet was resuspended in 6.5 µl 0.1x TE buffer and denatured by heating to 98°C for 2 min, then immediately placed on ice. The denatured DNA was centrifuged at 12,000 g for 30 s to collect all solution at the bottom of tube. Then, 1 µl AD2 (40 µM) and 7.5 µl of Instant Stick Ends Ligase Master Mix (New England Biolabs, M0370) were added. The mixture was kept overnight at 4 °C and purified with 0.8x volume AMPure XP (40 µl, 35 µl Milli-Q), then eluted with 16 µl 0.1x TE buffer. The eluted DNA was amplified using NEB Next Ultra II Q5 Master Mix with indexing primers for Illumina. The amplified products were purified by 0.9x AMPure XP beads and eluted with 25 µl 0.1x TE buffer. The concentration of eluted DNA was determined using Quantus Fluorometer (Promega). An equal amount of each library sample (≥ 20 ng) was pooled and purified again by 0.9x AMPure XP beads to remove residual primer-dimers, then eluted using 25 µl 10 mM Tris-HCl buffer (pH 8.0). The mixture was further diluted to 5 ng/µl and sent for sequencing. The pooled libraries were sequenced as 1x100 bp on an Illumina NovaSeq6000 sequencer.

Omni-ATAC-seq

An improved ATAC-seq method[1] was used to generate chromatin accessibility profiles in wildtype and ASH1L$^{-/-}$ cells. After UV-C irradiation at 20 J·m^{-2}, cells were allowed to recover for 3 h at 37°C in fresh medium or were processed directly in the case of unirradiated controls. To digest the DNA of dead or damaged cells before harvesting, cells were treated with deoxyribonuclease I (Sigma-Aldrich, D4513-1VL) at a final concentration of 200 units/mL and resuspended in Hanks' Balanced Salt Solution (Sigma-Aldrich, 55037C) for 30 min at 37°C. Cells were then washed three times with PBS, removed by trypsinization and counted. A cell viability of > 90% was confirmed by the addition of 0.4% (wt/vol) trypan blue solution (Thermo Fisher Scientific, 15-250-061).

Next, 50,000 cells were transferred to a 1.5-mL DNA LoBind tube (Eppendorf). Cells were pelleted at 2,500 g for 5 min at 4°C. The supernatant was removed and the cell pellet was resuspended in 50 µL of ice cold ATAC-seq lysis buffer [10 mM Tris-HCl, pH 7.4, 10 mM NaCl, 3 mM $MgCl_2$, 0.1% (wt/vol) NP-40, 0.1% (wt/vol) Tween 20, and 0.01% (wt/vol) digitonin] and left on ice for 3 min before adding 1 mL of ice-cold ATAC-seq wash buffer [10 mM Tris-HCl, pH 7.4, 10 mM NaCl, 3 mM $MgCl_2$, 0.1% (wt/vol) Tween 20]. Subsequently, nuclei were pelleted at 2,500 g for 10 min at 4 °C and the supernatant was aspirated. The nuclei were resuspended and exposed to the transposition reaction following the manufacturer's protocol (Diagenode, C01080002). This assay is based on the use of the transposase Tn5, which "tagments" the DNA by cleaving and tagging, through the ligation of sequencing adapters, accessible chromatin.

Transposition reactions were stopped by adding 5 volumes of DNA-binding buffer from the Zymo Concentrator-5 kit (Zymo Research, D4013). DNA was purified using this kit

according to the manufacturer's instructions including a 10,000 g centrifugation step. Samples were eluted into DNA LoBind tubes with 23 µL of DNA elution buffer pre-warmed to 50°C. After an incubation of 20 min and a 30-sec centrifugation, the eluate was re-added to the column, incubated for 5 min and centrifuged again for 30 sec.

The transposed DNA fragments were pre-amplified for 5 cycles with the LabCycler (SensoQuest) using Q5 High-Fidelity DNA Polymerase (New England Biolabs) and unique dual indices (primers are listed in Supplementary Table 4). After 5 cycles, samples were removed from the thermocycler and stored on ice. To avoid overamplification, qPCR was used to determine the number of additional cycles to run: 5 µL of the pre-amplified mixture were further amplified on the CFX384 Touch Real-Time PCR detection system (Bio-Rad). The required number of additional amplification cycles corresponded to the maximum relative fluorescence units divided by 4. This additional number of cycles was run using the remaining pre-amplified DNA. Thereafter, a double-sided clean-up with AMPure XP beads (Beckman Coulter, A63880) was performed. All steps were conducted at room temperature and in DNA LoBind tubes. To remove larger fragments (> 1,000 bp), 0.5 volumes of AMPure XP beads were thoroughly mixed with each sample by pipetting up and down 10 times. Samples were incubated for at least 20 min. Tubes were placed on a magnetic rack for 5 min before the supernatant was transferred to a new tube. Smaller fragments (75-100 bp) were removed by adding a 1.3-fold volume of AMPure XP beads, resulting in a final beads-to-sample ratio of 1.8. After a 20-min incubation, the tubes were placed on the magnetic rack for another 5 min. The supernatant was removed and the beads remaining in the tube were washed with freshly made 80% ethanol by pipetting ethanol over the beads 10 times. After the last wash step, ethanol was removed and the tubes were left on the magnetic rack for 10 min with the caps open to allow all residual ethanol to evaporate. Beads were resuspended in 20 µL Milli-Q water and again placed on the magnetic rack for 5 min. The supernatant was transferred to a new tube and the procedure was repeated once more before DNA concentration was determined using the Invitrogen Qubit 4 Fluorometer. To verify a desired fragment size of 200-1,000 base pairs, purified libraries were analyzed on the 4200 TapeStation system from Agilent using the High Sensitivity D1000 ScreenTape assay. Next, libraries were pooled at equimolar concentrations and sent to be sequenced to 50 million reads per sample on an Illumina Novaseq 6000 (SP flowcell) as 50-base pair reads.

Raw reads (FASTQ files) were processed based on the ENCODE ATAC-seq pipeline for paired-end reads. First, adapters were detected and trimmed (cutadapt v.1.9.1). Next, the trimmed reads were aligned (bowtie2 v.2.4.5) to the human reference genome build GCA hg38 excluding chromosome Y but including non-canonical contigs. These alignments underwent further filtering steps (samtools v.1.7, bedtools v.2.29.2, Picard v.2.23.8) to exclude reads that were PCR or optical duplicates, were unmapped or non-primary alignments, failed platform and/or vendor quality checks, had mapping quality scores below 30, or mapped to ENCODE blacklisted regions, non-canonical chromosomes or mitochondrial DNA. The filtered alignments were used to call peaks (macs2 v.2.2.7.1 with default parameters), which were used to verify replicability across biological replicates (idr v.2.0.4.2). ATAC-seq signal tracks were generated and uploaded to the IGV[2,3] to visualize continuous signals. Custom bash and R scripts were used to find the distribution of ATAC-seq peaks under the different conditions.

Mass spectrometry

The liquid chromatography/tandem mass spectrometry (LC-MS/MS) analysis of trypsin-treated peptides was performed on an Q Exactive mass spectrometer (Thermo Scientific) equipped with a Digital PicoView source (New Objective) and coupled to a nanoAcquity UPLC (Waters). Solvent composition at the two channels was 0.1% (vol/vol) formic acid for channel A and 0.1% formic acid, 99.9% (vol/vol) acetonitrile for channel B. Column

temperature was 50°C. For each sample, 2 μL of peptides were loaded on a commercial Symmetry C18 trap column (5 μm, 180 μm x 20 mm, Waters) connected to a BEH300 C18 column (1.7 μm, 75 μm x 150 m, Waters Inc.). The peptides were eluted at a flow rate of 300 nL/min with a gradient from 5 to 35% B in 60 min, 35 to 60% B in 5 min and 60 to 95% B in 10 min before equilibrating back to 5% B.

The mass spectrometer was operated in data-dependent mode (DDA). Full-scan MS spectra (350−1500 m/z) were acquired at a resolution of 70,000 at 200 m/z after accumulation to a target value of 3,000,000, followed by higher-energy collision dissociation (HCD) fragmentation on the twelve most intense signals per cycle. Ions were isolated with a 1.2 m/z isolation window and fragmented by HCD using a normalized collision energy of 25%. HCD spectra were acquired at a resolution of 35,000 and a maximum injection time of 120 ms. The automatic gain control (AGC) was set to 100,000 ions. Charge state screening was enabled and singly and unassigned charge states were rejected. Only precursors with intensity above 25,000 were selected for MS/MS. Precursor masses previously selected for MS/MS measurement were excluded from further selection for 40 s, and the exclusion window tolerance was set at 10 ppm. The samples were acquired using internal lock mass calibration on m/z 371.1010 and 445.1200. The mass spectrometry proteomics data were handled using the local laboratory information management system (LIMS)[4] and all relevant data have been deposited to the ProteomeXchange consortium via the PRIDE (http;//www.ebi.ac.uk/pride) repository.

The acquired raw MS data were processed by MaxQuant v.2.0.1.0, followed by protein identification using the integrated Andromeda search engine[5]. Spectra were searched against the Uniprot Homo sapiens reference proteome (taxonomy 9606, canonical version from 2019-07-09), concatenated to its reversed decoyed fasta database and common protein contaminants. Methionine oxidation and N-terminal protein acetylation were set as variable. Enzyme specificity was set to trypsin/P allowing a minimal peptide length of 7 amino acids and a maximum of two missed cleavages. MaxQuant Orbitrap default search settings were used with a fragment ion mass tolerance of 20 ppm and a parent ion tolerance of 10 ppm. The applied cutoffs entailed a minimal score for modified peptides of 40, and a minimal delta score for modified peptides of 6. The minimum number of unique peptides for protein identification was 2 peptides. The protein false discovery rate (FDR) was 1.0% and the peptide FDR was 0.1%.

Supplementary References

1. Corces, M. R. *et al.* An improved ATAC-seq protocol reduces background and enables interrogation of frozen tissues. *Nat. Methods* **14**, 959–962 (2017).
2. Robinson, J. T. *et al.* Integrative genomics viewer. *Nat. Biotechnol.* **29**, 24–26 (2011).
3. Thorvaldsdóttir, H., Robinson, J. T. & Mesirov, J. P. Integrative genomics viewer (IGV): high-performance genomics data visualization and exploration. *Brief. Bioinform.* **14**, 178–192 (2013).
4. Turker, C. *et al.* B-Fabric: The Swiss army knife for life sciences. In: Proceedings of the 13th International Conference on Extending Database Technology, Lausanne, Switzerland, 22-26 March 2010, 717–720 (2010).
5. Cox, J. & Mann, M. MaxQuant enables high peptide identification rates, individualized p.p.b.-range mass accuracies and proteome-wide protein quantification. *Nat. Biotechnol.* **26**, 1367–1372 (2008).
6. Martin, M. Cutadapt removes adapter sequences from high-throughput sequencing reads. *EMBnet Journal* **17**, 10-12 (2011).
7. Zhang, Y. *et al.* Model-based analysis of ChIP-seq (MACS). *Genome Biol.* **9**, R137 (2008).

8. Li, Q. *et al.* Measuring reproducibility of high-throughput experiments. *Annals of Applied Statistics* **5**, 1752-1779 (2011).
9. Bushnell, B. *et al.* BBMap: A fast, accurate, splice-aware aligner. *No. LBNL-7065E*. Ernest Orlando Lawrence Berkeley National Laboratory, Berkeley, CA. (2014).
10. Li, H. & Durbin, R. Fast and accurate long-read alignment with Burrows-Wheeler transform. *Bioinformatics* **26**, 589-595 (2010).
11. Li, H. *et al.* The sequence alignment/map format and SAMtools. *Bioinformatics* **25**, 2078-2079 (2009).
12. Quinlan, A.R. & Hall, I.M. BEDTools: a flexible suite of utilities for comparing genomic features. *Bioinformatics* **26**, 841-842 (2010).
13. R Core Team. R: a language and environment for statistical computing. R Foundation for Statistical Computing, Vienna, Austria. URL https://www.R-projct.org/ (2021).
14. Eddelbuettel, D. & François, R. Rcpp: seamless R and C++ integration. *Journal of Statistical Software* **40**, 1-18 (2011).
15. Robinson M.D. *et al.* edgeR: a Bioconductor package for differential expression analysis of digital gene expression data. *Bioinformatics* **26**, 139-140 (2010).
16. Love M.I. *et al.* Moderated estimation of fold change and dispersion for RNA-seq data with DESeq2. *Genome Biol*. **15**, 550 (2014).
17. Kassambara, A. ggpubr: 'ggplot2' based publication ready plots. [R package ggpubr veresion 0.4.0] (2020).
18. Wickham, H. ggplot2: elegant graphics for data analysis. S pringer-Verlag New York. ISBN 978-3-319-24277-4, https://ggplot2.tidyverse.org (2016).
19. Wickham, H. *et al.* dplyr: a grammar of data manipulation. https://dplyr.tidyverse.org, https://github.com/tidyverse/dplyr (2023).
20. Wickham, H. *et al.* tidyr: tidy messy data. https://tidyr.tidyverse.org, https://github.com/tidyverse/tidyr (2023).
21. Kassambra, A. rstatix: pipe-friendly framework for basic statistical tests. R package version 0.7.0. https://CRAN.R-project.org/package=rstatix (2021).
22. Langmead, B. & Salzberg, S. Fast-gapped read alignment with Bowtie 2. *Nat. Methods* **9**, 357-359 (2012).
23. Cox, J. *et al.* Andromeda: a peptide search engine integrated into the MaxQuant environment. *J. Proteome Res.* **10**, 1794-1805 (2011).
24. Schneider, C.A. *et al.* NIH Image to ImageJ: 25 years of image analysis. *Nat. Methods* **9**, 671-675 (2012).
25. FlowJoTM Software for Mac [software application] Version 10.9. Ashland, OR: Becton, Dickinson and Company; 2023.
