# Supplementary material for: ASH1L-MRG15 methyltransferase deposits H3K4me3 and FACT for damage verification in nucleotide excision repair
Source: Nat Commun. 2023 Jul 1;14:3892. doi: 10.1038/s41467-023-39635-7 (PMC10314917; doi:10.1038/s41467-023-39635-7)
Supplement: Supplementary file 3 — Reporting Summary [file 41467_2023_39635_MOESM3_ESM.pdf]

nature portfolio

Corresponding author(s): Hanspeter Naegeli

Last updated by author(s): Jun 9, 2023

Reporting Summary

Nature Portfolio wishes to improve the reproducibility of the work that we publish. This form provides structure for consistency and transparency in reporting. For further information on Nature Portfolio policies, see our Editorial Policies and the Editorial Policy Checklist.

Statistics

For all statistical analyses, confirm that the following items are present in the figure legend, table legend, main text, or Methods section.

n/a	Confirmed	
☐	☒	The exact sample size (*n*) for each experimental group/condition, given as a discrete number and unit of measurement
☐	☒	A statement on whether measurements were taken from distinct samples or whether the same sample was measured repeatedly
☐	☒	The statistical test(s) used AND whether they are one- or two-sided<br>*Only common tests should be described solely by name; describe more complex techniques in the Methods section.*
☒	☐	A description of all covariates tested
☒	☐	A description of any assumptions or corrections, such as tests of normality and adjustment for multiple comparisons
☐	☒	A full description of the statistical parameters including central tendency (e.g. means) or other basic estimates (e.g. regression coefficient) AND variation (e.g. standard deviation) or associated estimates of uncertainty (e.g. confidence intervals)
☐	☒	For null hypothesis testing, the test statistic (e.g. *F*, *t*, *r*) with confidence intervals, effect sizes, degrees of freedom and *P* value noted<br>*Give P values as exact values whenever suitable.*
☒	☐	For Bayesian analysis, information on the choice of priors and Markov chain Monte Carlo settings
☒	☐	For hierarchical and complex designs, identification of the appropriate level for tests and full reporting of outcomes
☒	☐	Estimates of effect sizes (e.g. Cohen's *d*, Pearson's *r*), indicating how they were calculated

Our web collection on statistics for biologists contains articles on many of the points above.

Software and code

Policy information about availability of computer code

Data collection	Image Studio v.5.2.5, ImageJ v.2.8.0, FlowJo v10.9
Data analysis	GraphPad Prism 9.5.0, SMARTselection algorithm (version pSeven 6.16), ENCODE ChIP-seq pipeline, TrimGalore v.0.6.5, macs2 v.2.2.7.1, idr v.2.0.4.2, CPDSeqer pipeline, bbmap v.38.90, bwa mem v.07.17-r1188, samtools v.1.7, bedtools v.2.29.2, Picard v.2.23.8. R packages (R v.4.0.3 and 4.1.0): Rcpp v.1.0.8.3, edgeR v.3.34.1, DESeq2 v.1.32.0, ggpubr v.0.4.0, ggplot2 v.3.3.5, dplyr v.1.0.8, tidyr v.1.2.0, rstatix v.0.7.0; see below for a complete description of the software used for ChIP analyses. ATAC-seq-specific tools (mentioned in Supplementary Info): cutadapt v.1.9.1, bowtie2 v.2.4.5. Mass spectrometry analysis tools (provided in Supplementary Info): MaxQuant v.2.0.1.0; Andromeda search engine.

For manuscripts utilizing custom algorithms or software that are central to the research but not yet described in published literature, software must be made available to editors and reviewers. We strongly encourage code deposition in a community repository (e.g. GitHub). See the Nature Portfolio guidelines for submitting code & software for further information.

Data

Policy information about availability of data

All manuscripts must include a data availability statement. This statement should provide the following information, where applicable:

- Accession codes, unique identifiers, or web links for publicly available datasets
- A description of any restrictions on data availability
- For clinical datasets or third party data, please ensure that the statement adheres to our policy

The genomics data generated in this study have been deposited in NCBI's Gene Expression Omnibus database under GEO Series accession code GSE227009 (https://www.ncbi.nlm.nih.gov/geo/query/acc.cgi?acc=GSE227009). Non-sequencing data generated in this study (including the mass spectrometry raw data) are available in the OSF database (DOI 10.17605/OSF.IO/WVR9C; https://osf.io/wvr9c). Additionally, all MS data are available at the ProteomeXchange consortium via the PRIDE (http://www.ebi.ac.uk/pride) partner repository with the data set identifier PXD043089. The data used for graphical displays are provided in the Source Data file. Sequencing reads were aligned to the GRCh38 or hg38 build of the human genome available from NCBI (https://www.ncbi.nlm.nih.gov/assembly/?term=GCA_000001405).

Human research participants

Policy information about studies involving human research participants and Sex and Gender in Research.

Reporting on sex and gender	N/A
Population characteristics	N/A
Recruitment	N/A
Ethics oversight	N/A

Note that full information on the approval of the study protocol must also be provided in the manuscript.

Field-specific reporting

Please select the one below that is the best fit for your research. If you are not sure, read the appropriate sections before making your selection.

☒ Life sciences ☐ Behavioural & social sciences ☐ Ecological, evolutionary & environmental sciences

For a reference copy of the document with all sections, see nature.com/documents/nr-reporting-summary-flat.pdf

Life sciences study design

All studies must disclose on these points even when the disclosure is negative.

Sample size	A minimum of three biologically independent experiments were conducted to perform statistical analyses. In most cases, 4-5 replicates were provided to consolidate the findings. Exceptions: genome-wide DNA-damage seq assay, whereby a single biological experiment provided sufficient data for statistical analyses; K36me2 ChIP-seq and ATAC-seq data, for which two independent biological replicates per condition were obtained.
Data exclusions	None
Replication	All replication experiments were successful and confirmed the hypothesis generated by the initial pilot experiment. Damage-seq data were replicated once in wildtype cells; all other experiments except the ATAC-seq and H3K36me2 ChIP-seq were performed in at least 3 independent biological replicates, as noted above under "Sample size." ChIP-seq and ATAC-seq replicability was verified by calculating the irreproducibility discovery rate of called peaks, as described under "Methodology- Replicability.
Randomization	Identical biological samples were randomly distributed to be subjected to the different experimental treatments. Co-variates were excluded by the use of internal (negative and positive) controls.
Blinding	Blinding was applied to all genome-wide sequencing experiments. Due to the low number of scientists involved, blinding was not possible for the hypothesis-driven experiments. However, each hypothesis-driven experiment was validated by the inclusion of negative and positive controls, such that blinding would not add any additional certainty to the outcome.

Reporting for specific materials, systems and methods

We require information from authors about some types of materials, experimental systems and methods used in many studies. Here, indicate whether each material, system or method listed is relevant to your study. If you are not sure if a list item applies to your research, read the appropriate section before selecting a response.

Materials & experimental systems

n/a	Involved in the study
☐	☒ Antibodies
☐	☒ Eukaryotic cell lines
☒	☐ Palaeontology and archaeology
☒	☐ Animals and other organisms
☒	☐ Clinical data
☒	☐ Dual use research of concern

Methods

n/a	Involved in the study
☐	☒ ChIP-seq
☐	☒ Flow cytometry
☒	☐ MRI-based neuroimaging

Antibodies

Antibodies used: The antibodies and their uses are described in detail in the Supplementary Table 2.

Validation: All antibodies were of commercial sources and validated by the manufacturer for immunoprecipitation, immunoblotting and in situ immunofluorescence studies (as appropriate) using negative controls. In addition, we validated the specificity of the antibodies in Western blots and immunofluorescence studies using knockdown approaches and our own knockdown cells as negative controls.

Eukaryotic cell lines

Policy information about cell lines and Sex and Gender in Research

Cell line source(s): HeLa, U2OS and HEK293T cells are from American Type Culture Collection (ATCC).

Authentication: Authentication was performed by short tandem repeat profiling

Mycoplasma contamination: The cell lines were tested negative for mycoplasma contamination

Commonly misidentified lines (See ICLAC register): None

ChIP-seq

Data deposition

☒ Confirm that both raw and final processed data have been deposited in a public database such as GEO.

☒ Confirm that you have deposited or provided access to graph files (e.g. BED files) for the called peaks.

Data access links
May remain private before publication.

Deposited data: https://www.ncbi.nlm.nih.gov/geo/query/acc.cgi?acc=GSE227009

Files in database submission

Raw ChIP-seq data:
H3K4me3_WT_noUV_IP_Rep1.R1.fastq.gz, H3K4me3_WT_noUV_IN_Rep1.R1.fastq.gz,
H3K4me3_WT_noUV_IP_Rep2.R1.fastq.gz, H3K4me3_WT_noUV_IN_Rep2.R1.fastq.gz,
H3K4me3_WT_noUV_IP_Rep3.R1.fastq.gz, H3K4me3_WT_noUV_IN_Rep3.R1.fastq.gz,
H3K4me3_WT_1h_IP_Rep1.R1.fastq.gz, H3K4me3_WT_1h_IN_Rep1.R1.fastq.gz, H3K4me3_WT_1h_IP_Rep2.R1.fastq.gz,
H3K4me3_WT_1h_IN_Rep2.R1.fastq.gz, H3K4me3_WT_1h_IP_Rep3.R1.fastq.gz, H3K4me3_WT_1h_IN_Rep3.R1.fastq.gz,
H3K4me3_WT_3h_IP_Rep1.R1.fastq.gz, H3K4me3_WT_3h_IN_Rep1.R1.fastq.gz, H3K4me3_WT_3h_IP_Rep2.R1.fastq.gz,
H3K4me3_WT_3h_IN_Rep2.R1.fastq.gz, H3K4me3_WT_3h_IP_Rep3.R1.fastq.gz, H3K4me3_WT_3h_IN_Rep3.R1.fastq.gz,
H3K4me3_ALKO_noUV_IP_Rep1.R1.fastq.gz, H3K4me3_ALKO_noUV_IN_Rep1.R1.fastq.gz,
H3K4me3_ALKO_noUV_IP_Rep2.R1.fastq.gz, H3K4me3_ALKO_noUV_IN_Rep2.R1.fastq.gz,
H3K4me3_ALKO_noUV_IP_Rep3.R1.fastq.gz, H3K4me3_ALKO_noUV_IN_Rep3.R1.fastq.gz,
H3K4me3_ALKO_1h_IP_Rep1.R1.fastq.gz, H3K4me3_ALKO_1h_IN_Rep1.R1.fastq.gz,
H3K4me3_ALKO_1h_IP_Rep2.R1.fastq.gz, H3K4me3_ALKO_1h_IN_Rep2.R1.fastq.gz,
H3K4me3_ALKO_1h_IP_Rep3.R1.fastq.gz, H3K4me3_ALKO_1h_IN_Rep3.R1.fastq.gz,
H3K4me3_ALKO_3h_IP_Rep1.R1.fastq.gz, H3K4me3_ALKO_3h_IN_Rep1.R1.fastq.gz,
H3K4me3_ALKO_3h_IP_Rep2.R1.fastq.gz, H3K4me3_ALKO_3h_IN_Rep2.R1.fastq.gz,
H3K4me3_ALKO_3h_IP_Rep3.R1.fastq.gz, H3K4me3_ALKO_3h_IN_Rep3.R1.fastq.gz, XPC_WT_noUV_IP_Rep1.R1.fastq.gz,
XPC_WT_noUV_IP_Rep2.R1.fastq.gz, XPC_WT_noUV_IP_Rep3.R1.fastq.gz, XPC_WT_noUV_IP_Rep4.R1.fastq.gz,
XPC_WT_1h_IP_Rep1.R1.fastq.gz, XPC_WT_1h_IP_Rep2.R1.fastq.gz, XPC_WT_1h_IP_Rep3.R1.fastq.gz,
XPC_WT_3h_IP_Rep1.R1.fastq.gz, XPC_WT_3h_IP_Rep2.R1.fastq.gz, XPC_WT_3h_IP_Rep3.R1.fastq.gz,
XPC_ALKO_noUV_IP_Rep1.R1.fastq.gz, XPC_ALKO_noUV_IP_Rep2.R1.fastq.gz, XPC_ALKO_noUV_IP_Rep3.R1.fastq.gz,
XPC_ALKO_1h_IP_Rep1.R1.fastq.gz, XPC_ALKO_1h_IP_Rep2.R1.fastq.gz, XPC_ALKO_1h_IP_Rep3.R1.fastq.gz,
XPC_ALKO_3h_IP_Rep1.R1.fastq.gz, XPC_ALKO_3h_IP_Rep2.R1.fastq.gz, XPC_ALKO_3h_IP_Rep3.R1.fastq.gz,
XPC_XPCKO_noUV_IP_Rep1.R1.fastq.gz, XPC_XPCKO_noUV_IP_Rep2.R1.fastq.gz, XPC_XPCKO_1h_IP_Rep1.R1.fastq.gz,

XPC_XPCKO_1h_IP_Rep2.R1.fastq.gz, XPC_XPCKO_3h_IP_Rep1.R1.fastq.gz, XPC_XPCKO_3h_IP_Rep2.R1.fastq.gz, ASH1LCTD_ALKO_3h_Rep1_R1.fastq.gz, ASH1LCTD_ALKO_3h_Rep2_R1.fastq.gz, ASH1LCTD_ALKO_3h_Rep3_R1.fastq.gz, ASH1LCTD_ALKO_noUV_Rep1_R1.fastq.gz, ASH1LCTD_ALKO_noUV_Rep2_R1.fastq.gz, ASH1LCTD_ALKO_noUV_Rep3_R1.fastq.gz, EmptyVector_ALKO_3h_Rep1_R1.fastq.gz, EmptyVector_ALKO_3h_Rep2_R1.fastq.gz, EmptyVector_ALKO_3h_Rep3_R1.fastq.gz, EmptyVector_ALKO_noUV_Rep1_R1.fastq.gz, EmptyVector_ALKO_noUV_Rep2_R1.fastq.gz, EmptyVector_ALKO_noUV_Rep3_R1.fastq.gz, H3K36me2_WT_3h_IN_Rep1.R1.fastq.gz, H3K36me2_WT_3h_IN_Rep2.R1.fastq.gz, H3K36me2_WT_3h_IP_Rep1.R1.fastq.gz, H3K36me2_WT_3h_IP_Rep2.R1.fastq.gz, H3K36me2_WT_noUV_IN_Rep1.R1.fastq.gz, H3K36me2_WT_noUV_IN_Rep2.R1.fastq.gz, H3K36me2_WT_noUV_IP_Rep1.R1.fastq.gz, H3K36me2_WT_noUV_IP_Rep2.R1.fastq.gz, H3K36me2_supplementreads_WT_3h_IN_Rep1_R1.fastq.gz, H3K36me2_supplementreads_WT_3h_IN_Rep1_R2.fastq.gz, H3K36me2_supplementreads_WT_3h_IN_Rep2_R1.fastq.gz, H3K36me2_supplementreads_WT_3h_IN_Rep2_R2.fastq.gz, H3K36me2_supplementreads_WT_3h_IP_Rep1_R1.fastq.gz, H3K36me2_supplementreads_WT_3h_IP_Rep1_R2.fastq.gz, H3K36me2_supplementreads_WT_3h_IP_Rep2_R1.fastq.gz, H3K36me2_supplementreads_WT_3h_IP_Rep2_R2.fastq.gz, H3K36me2_supplementreads_WT_noUV_IN_Rep1_R1.fastq.gz, H3K36me2_supplementreads_WT_noUV_IN_Rep1_R2.fastq.gz, H3K36me2_supplementreads_WT_noUV_IN_Rep2_R1.fastq.gz, H3K36me2_supplementreads_WT_noUV_IN_Rep2_R2.fastq.gz, H3K36me2_supplementreads_WT_noUV_IP_Rep1_R1.fastq.gz, H3K36me2_supplementreads_WT_noUV_IP_Rep1_R2.fastq.gz, H3K36me2_supplementreads_WT_noUV_IP_Rep2_R1.fastq.gz, H3K36me2_supplementreads_WT_noUV_IP_Rep2_R2.fastq.gz

Processed ChIP-seq files:
bigWigs:
H3K4me3_WT_noUV_Rep1.bw, H3K4me3_WT_noUV_Rep2.bw, H3K4me3_WT_noUV_Rep3.bw, H3K4me3_WT_1h_Rep1.bw, H3K4me3_WT_1h_Rep2.bw, H3K4me3_WT_1h_Rep3.bw, H3K4me3_WT_3h_Rep1.bw, H3K4me3_WT_3h_Rep2.bw, H3K4me3_WT_3h_Rep3.bw, H3K4me3_ALKO_noUV_Rep1.bw, H3K4me3_ALKO_noUV_Rep2.bw, H3K4me3_ALKO_noUV_Rep3.bw, H3K4me3_ALKO_1h_Rep1.bw, H3K4me3_ALKO_1h_Rep2.bw, H3K4me3_ALKO_1h_Rep3.bw, H3K4me3_ALKO_3h_Rep1.bw, H3K4me3_ALKO_3h_Rep2.bw, H3K4me3_ALKO_3h_Rep3.bw, XPC_WT_noUV_Rep1.bw, XPC_WT_noUV_Rep2.bw, XPC_WT_noUV_Rep3.bw, XPC_WT_noUV_Rep4.bw, XPC_WT_1H_Rep1.bw, XPC_WT_1H_Rep2.bw, XPC_WT_1H_Rep3.bw, XPC_WT_3H_Rep1.bw, XPC_WT_3H_Rep2.bw, XPC_WT_3H_Rep3.bw, XPC_ALKO_noUV_Rep1.bw, XPC_ALKO_noUV_Rep2.bw, XPC_ALKO_noUV_Rep3.bw, XPC_ALKO_1H_Rep1.bw, XPC_ALKO_1H_Rep2.bw, XPC_ALKO_1H_Rep3.bw, XPC_ALKO_3H_Rep1.bw, XPC_ALKO_3H_Rep2.bw, XPC_ALKO_3H_Rep3.bw, ASH1LCTD_ALKO_3h_Rep1.bw, ASH1LCTD_ALKO_3h_Rep2.bw, ASH1LCTD_ALKO_3h_Rep3.bw, ASH1LCTD_ALKO_noUV_Rep1.bw, ASH1LCTD_ALKO_noUV_Rep2.bw, ASH1LCTD_ALKO_noUV_Rep3.bw, H3K36me2_WT_3h_Rep1.bw, H3K36me2_WT_3h_Rep2.bw, H3K36me2_WT_noUV_Rep1.bw, H3K36me2_WT_noUV_Rep2.bw

Peak files:
H3K4me3_WT_noUV_Rep1.narrowPeak.gz, H3K4me3_WT_noUV_Rep2.narrowPeak.gz, H3K4me3_WT_noUV_Rep3.narrowPeak.gz, H3K4me3_WT_1h_Rep1.narrowPeak.gz, H3K4me3_WT_1h_Rep2.narrowPeak.gz, H3K4me3_WT_1h_Rep3.narrowPeak.gz, H3K4me3_WT_3h_Rep1.narrowPeak.gz, H3K4me3_WT_3h_Rep2.narrowPeak.gz, H3K4me3_WT_3h_Rep3.narrowPeak.gz, H3K4me3_ALKO_noUV_Rep1.narrowPeak.gz, H3K4me3_ALKO_noUV_Rep2.narrowPeak.gz, H3K4me3_ALKO_noUV_Rep3.narrowPeak.gz, H3K4me3_ALKO_1h_Rep1.narrowPeak.gz, H3K4me3_ALKO_1h_Rep2.narrowPeak.gz, H3K4me3_ALKO_1h_Rep3.narrowPeak.gz, H3K4me3_ALKO_3h_Rep1.narrowPeak.gz, H3K4me3_ALKO_3h_Rep2.narrowPeak.gz, H3K4me3_ALKO_3h_Rep3.narrowPeak.gz, XPC_WT_noUV_Rep1.broadPeak.gz, XPC_WT_noUV_Rep2.broadPeak.gz, XPC_WT_noUV_Rep3.broadPeak.gz, XPC_WT_noUV_Rep4.broadPeak.gz, XPC_WT_1H_Rep1.broadPeak.gz, XPC_WT_1H_Rep2.broadPeak.gz, XPC_WT_1H_Rep3.broadPeak.gz, XPC_WT_3H_Rep1.broadPeak.gz, XPC_WT_3H_Rep2.broadPeak.gz, XPC_WT_3H_Rep3.broadPeak.gz, XPC_ALKO_noUV_Rep1.broadPeak.gz, XPC_ALKO_noUV_Rep2.broadPeak.gz, XPC_ALKO_noUV_Rep3.broadPeak.gz, XPC_ALKO_1H_Rep1.broadPeak.gz, XPC_ALKO_1H_Rep2.broadPeak.gz, XPC_ALKO_1H_Rep3.broadPeak.gz, XPC_ALKO_3H_Rep1.broadPeak.gz, XPC_ALKO_3H_Rep2.broadPeak.gz, XPC_ALKO_3H_Rep3.broadPeak.gz, ASH1LCTD_ALKO_3h_Rep1.broadPeak.gz, ASH1LCTD_ALKO_3h_Rep2.broadPeak.gz, ASH1LCTD_ALKO_3h_Rep3.broadPeak.gz, ASH1LCTD_ALKO_noUV_Rep1.broadPeak.gz, ASH1LCTD_ALKO_noUV_Rep2.broadPeak.gz, ASH1LCTD_ALKO_noUV_Rep3.broadPeak.gz, H3K36me2_WT_3h_Rep1.broadPeak.gz, H3K36me2_WT_3h_Rep2.broadPeak.gz, H3K36me2_WT_noUV_Rep1.broadPeak.gz, H3K36me2_WT_noUV_Rep2.broadPeak.gz

Genome browser session
(e.g. UCSC)

http://genome.ucsc.edu/s/myancoskie/ASH1Lmanuscript_ChIP
Due to limited resources available to host publicly accessible signal tracks for shareable genome browser sessions, we provide H3K4me3, H3K36me2, XPC, and ASH1L-CTD ChIP-seq signal tracks of an 8 megabase-pair region of chromosome 20 (hg38 chr20:40,283,000-40,291,000) which encompasses the coordinates depicted in the Figure 3c genome browser screenshot.

Methodology

Replicates

For H3K4me3 ChIP-seq, a separate input control was prepared for each IP. Samples were prepared in triplicate, for a total of 18 IP and 18 input controls. Replicability was verified using the IDR (irreproducibility discovery rate) tool on peaks called from true, pooled, self-pseudo, and pooled pseudo-replicates. Because self-consistency and rescue ratios never exceeded a factor of 2, all replicates were retained. Input controls were pooled to calculate fold change, as they met the ENCODE ChIP-seq pipleine checklist for pooling. For XPC ChIP-seq, WT non-UV IP samples were prepared in quadruplicate, XPC knockout IPs in duplicate, and all other cell types and

timepoints in triplicate. Replicability was verified with IDR as previously described, with all replicates retained in the analysis due to passing self-consistency and rescue ratios obtained from IDR. As XPC is genreally constitutively bound to chromatin, XPC knockout IP samples were used as controls, instead of the usual input controls, following the "Knockout implemented normalization" concept from Krebs et al. 2014. The XPC knockout IPs were pooled as per ENCODE pipeline recommendations. For H3K36me2 ChIP-seq added to the revised manuscript, ChIP-seq was performed on biological duplicates, with a separate input control prepared for each IP and with input controls pooled in the pipeline and with IDR used to verify replicability. For ASH-CTD ChIP-seq added to the revised manuscript, ChIP-seq was performed in triplicate. A separate input control (empty vector transfected into ASH1L knockout cells) was prepared for each IP (ASH1L-CTD transfected into ASH1L knockout cells); input controls were pooled for the 3H timepoint but left unpooled for the nonUV timepoint following ENCODE specifications.

Sequencing depth

All ChIP-seq samples were sequenced as single-end 100 base pair reads. For the H3K36me2 ChIP-seq, an additional 5-8 million 150 bp read pairs were obtained so as to meet ENCODE standards of at least 45 million useable mapped fragments per replicate for a histone mark with a broad peak distribution; these were processed as paired-end until merging with single-end reads at the end of the pipeline, just prior to calling peaks and generating signal fold tracks. Total read counts (in order of FASTQ file names provided above):

H3K4me3 samples: 51366612 (WT noUV IP Rep1), 56878635 (WT noUV IN Rep1), 76994239 (WT noUV IP Rep2), 48077438 (WT noUV IN Rep2), 65918462 (WT noUV IP Rep3), 50995535 (WT noUV IN Rep3), 66315006 (WT 1H IP Rep1), 47579914 (WT 1H IN Rep1), 69514394 (WT 1H IP Rep2), 57895860 (WT 1H IN Rep2), 83774559 (WT 1H IP Rep3), 53021456 (WT 1H IN Rep3), 80044306 (WT 3H IP Rep1), 58960739 (WT 3H IN Rep1), 48710313 (WT 3H IP Rep2), 50943217 (WT 3H IN Rep2), 77084251 (WT 3H IP Rep3), 46889050 (WT 3H IN Rep3), 63277703 (ALKO noUV IP Rep1), 61024253 (ALKO noUV IN Rep1), 63638338 (ALKO noUV IP Rep2), 61909180 (ALKO noUV IN Rep2), 63969954 (ALKO noUV IP Rep3), 71278862 (ALKO noUV IN Rep3), 64317270 (ALKO 1H IP Rep1), 59682750 (ALKO 1H IN Rep1), 63181543 (ALKO 1H IP Rep2), 45953033 (ALKO 1H IN Rep2), 68288922 (ALKO 1H IP Rep3), 59892993 (ALKO 1H IN Rep3), 71115278 (ALKO 3H IP Rep1), 65186018 (ALKO 3H IN Rep1), 59163745 (ALKO 3H IP Rep2), 56461403 (ALKO 3H IN Rep2), 66534500 (ALKO 3H IP Rep3), 57939958 (ALKO 3H IN Rep3).

XPC samples: 111135914 (WT noUV Rep1), 59818316 (WT noUV Rep2), 58819533 (WT noUV Rep3), 27160860 (WT noUV Rep4), 115136526 (WT 1H Rep1), 41443635 (WT 1H Rep2), 68473642 (WT 1H Rep3), 85854229 (WT 3H Rep1), 54562468 (WT 3H Rep2), 74937375 (WT 3H Rep3), 59910695 (ALKO noUV Rep1), 66774942 (ALKO noUV Rep2), 103843778 (ALKO noUV Rep3), 62282716 (ALKO 1H Rep1), 47607959 (ALKO 1H Rep2), 82139400 (ALKO 1H Rep3), 104979600 (ALKO 3H Rep1), 44699310 (ALKO 3H Rep2), 92914833 (ALKO 3H Rep3), 113577736 (XPCKO noUV Rep1), 25864471 (XPCKO noUV Rep2), 100030554 (XPCKO 1H Rep1), 27090334 (XPCKO 1H Rep2), 43374190 (XPCKO 3H Rep1), 27557142 (XPCKO 3H Rep2).

ASH1L-CTD samples:
45407275 (ASH1L-CTD ALKO 3H Rep1), 48730068 (ASH1L-CTD ALKO 3H Rep2), 43933565 (ASH1L-CTD ALKO 3H Rep3), 48817283 (ASH1L-CTD ALKO noUV Rep1), 46923680 (ASH1L-CTD ALKO noUV Rep2), 44774965 (ASH1L-CTD ALKO noUV Rep3), 53808745 (Empty Vector ALKO 3H Rep1), 44520793 (Empty Vector ALKO 3H Rep2), 49060714 (Empty Vector ALKO 3H Rep3), 53704875 (Empty Vector ALKO noUV Rep1), 50346263 (Empty Vector ALKO noUV Rep2), 46403799 (Empty Vector ALKO noUV Rep3).

H3K36me2 samples:
38918074 (WT 3H IN Rep1), 42889009 (WT 3H IN Rep2), 47785712 (WT 3H IP Rep1), 53474617 (WT 3H IP Rep2), 45446408 (WT noUV IN Rep1), 43718922 (WT noUV IN Rep2), 47158110 (WT noUV IP Rep1), 44985534 (WT noUV IP Rep2), 5868143 (WT 3H IN Rep1 supplemental read pairs), 6292239 (WT 3H IN Rep2 supplemental read pairs), 7103259 (WT 3H IP Rep1 supplemental read pairs), 7822314 (WT 3H IP Rep2 supplemental read pairs), 7189719 (WT noUV IN Rep1 supplemental read pairs), 6773169 (WT noUV IN Rep2 supplemental read pairs), 7116126 (WT noUV IP Rep1 supplemental read pairs), 7074767 (WT noUV IP Rep2 supplemental read pairs).

Uniquely mapped reads (in order of FASTQ file names provided above; where "uniquely mapping" is defined as reads having a mapping quality of 30 or better, having removed blacklisted reads based on the Kundaje lab ChIP-seq blacklist and having removed reads under the SAM flag 1804 - and additionally for the H3K36me2 paired-end supplemental reads, having retained properly paired reads with SAM flag 2):
H3K4me3 samples: 42285223 (WT noUV IP Rep1), 46396545 (WT noUV IN Rep1), 63179374 (WT noUV IP Rep2), 29592514 (WT noUV IN Rep2), 53641772 (WT noUV IP Rep3), 33227118 (WT noUV IN Rep3), 54425731 (WT 1H IP Rep1), 37230876 (WT 1H IN Rep1), 56412749 (WT 1H IP Rep2), 36726195 (WT 1H IN Rep2), 67495699 (WT 1H IP Rep3), 33220371 (WT 1H IN Rep3), 63661195 (WT 3H IP Rep1), 38871949 (WT 3H IN Rep1), 39172989 (WT 3H IP Rep2), 34833686 (WT 3H IN Rep2), 61782750 (WT 3H IP Rep3), 29718145 (WT 3H IN Rep3), 52755062 (ALKO noUV IP Rep1), 42116291 (ALKO noUV IN Rep1), 52692230 (ALKO noUV IP Rep2), 40370156 (ALKO noUV IN Rep2), 52967251 (ALKO noUV IP Rep3), 45551581 (ALKO noUV IN Rep3), 54322999 (ALKO 1H IP Rep1), 45794983 (ALKO 1H IN Rep1), 51539605 (ALKO 1H IP Rep2), 29380835 (ALKO 1H IN Rep2), 55949461 (ALKO 1H IP Rep3), 38616436 (ALKO 1H IN Rep3), 58020232 (ALKO 3H IP Rep1), 52883382 (ALKO 3H IN Rep1), 49017969 (ALKO 3H IP Rep2), 38890669 (ALKO 3H IN Rep2), 54378262 (ALKO 3H IP Rep3), 39251786 (ALKO 3H IN Rep3).

XPC samples: 95102706 (WT noUV Rep1), 51633943 (WT noUV Rep2), 50705732 (WT noUV Rep3), 23353677 (WT noUV Rep4), 98098927 (WT 1H Rep1), 35868526 (WT 1H Rep2), 58927039 (WT 1H Rep3), 72816568 (WT 3H Rep1), 47041432 (WT 3H Rep2), 64607832 (WT 3H Rep3), 51772433 (ALKO noUV Rep1), 57860070 (ALKO noUV Rep2), 89948057 (ALKO noUV Rep3), 53560262 (ALKO 1H Rep1), 41107719 (ALKO 1H Rep2), 71093461 (ALKO 1H Rep3), 89738269 (ALKO 3H Rep1), 38550556 (ALKO 3H Rep2), 80242955 (ALKO 3H Rep3), 92167348 (XPCKO noUV Rep1), 22341970 (XPCKO noUV Rep2), 14475620 (XPCKO 1H Rep1), 23361756 (XPCKO 1H Rep2), 37695578 (XPCKO 3H Rep1), 23936675 (XPCKO 3H Rep2).

ASH1L-CTD samples: 38501421 (ASH1L-CTD ALKO 3H Rep1), 41492501 (ASH1L-CTD ALKO 3H Rep2), 37215376 (ASH1L-CTD ALKO 3H Rep3), 41480859 (ASH1L-CTD ALKO noUV Rep1), 39175699 (ASH1L-CTD ALKO noUV Rep2), 38247509 (ASH1L-CTD ALKO noUV Rep3), 45193078 (Empty Vector ALKO 3H Rep1), 37232559 (Empty Vector ALKO 3H Rep2), 41011283 (Empty Vector ALKO 3H Rep3), 45423198 (Empty Vector ALKO noUV Rep1), 42758743 (Empty Vector ALKO noUV Rep2), 38794082 (Empty Vector ALKO noUV Rep3).

H3K36me2 samples: 33700177 (WT 3H IN Rep1),41805665 (WT 3H IP Rep1),37172153 (WT 3H IN Rep2),47314135 (WT 3H IP Rep2),39424811 (WT noUV IN Rep1),41962781 (WT noUV IP Rep1),37907614 (WT noUV IN Rep2),40008802 (WT noUV IP Rep2), 10569886 (WT 3H IN Rep1 supplemental read pairs), 12957463 (WT 3H IP Rep1 supplemental read pairs), 11345624 (WT 3H IN Rep2

supplemental read pairs), 14356176 (WT 3H IP Rep2 supplemental read pairs), 13034147 (WT noUV IN Rep1 supplemental read pairs), 13092040 (WT noUV IP Rep1 supplemental read pairs), 12227046 (WT noUV IN Rep2 supplemental read pairs), 13036432 (WT noUV IP Rep2 supplemental read pairs.

Antibodies

The antibodies and their uses are listed in Supplementary Table 1

Peak calling parameters

Reads were aligned (bwa mem v.0.7.17-r1188) to the human reference genome build GCA hg38 lacking chrY but including non-canonical contigs. Alignments were filtered to exclude reads with mapping quality scores below 30 (bedtools v.2.29.2), PCR and optical duplicates (Picard MarkDuplicates, v.2.23.8), ENCODE-blacklisted regions, non-canonical contigs, and unmapped or secondary alignments (samtools v.1.7). Filtered alignments were used to call peaks (macs2 v.2.2.7.1 with default parameters) and to calculate fragment extension size using custom R scripts (R v.4.0.3)72,73. Peaks were called using a p-value cutoff of 1e-2. Only robust peaks found in a majority of biological replicates were used in downstream analyses. For H3K4me3, peaks were called from the fold enrichment of each IP over each corresponding pooled input control. For XPC as previously discussed, peaks were called from the fold enrichment of each IP over each corresponding (pooled from 2 duplicates) XPC knockout cell IP. For ASH-CTD, peaks were called from the fold enrichment of each ASH1L-transfected IP over each corresponding (pooled for 3h timepoints; unpooled for non-UV timepoints) input controls, where input controls were prepared in triplicate from ASH1L knockout cells transfected with empty vector. For H3K36me2, peaks were called from the fold enrichment of IP (in duplicate) over (pooled from duplicate) input control. The following command was used to call peaks for both types of ChIP-seq experiments, with the XPC, ASH-CTD, and H3K36me2 ChIP-seq additionally including the parameter "--broad" due to their binding tending to have more of a broad than a narrow peak shape: macs2 callpeak -t <treatment tagAlign file> -c <control tagAlign file> -f BED -g hs -p 1e-2 --nomodel --shift 0 --extsize <fragment extension size from cross-correlation analyses> --keep-dup all -B --SPMR.

Data quality

Raw reads were trimmed to remove reads shorter than 99 base pairs and crop reads exceeding 101 bp (or for the H3K36me2 supplemental read pairs, to crop to 149-151 bp. Alignments were filtered to exclude reads with mapping quality scores below 30, PCR and optical duplicates, ENCODE-blacklisted regions, non-canonical contigs, unmapped or secondary alignments, and (for H3K36me2 supplementary read pairs) unpaired reads. Replicability across biological replicates was verified by calling peaks on true, pooled, self-pseudo, and pooled pseudo-replicates and checking with IDR (irreproducibility discovery rate) that rescue and self-consistency ratios were below a factor of 2. Peaks were called using a p-value cutoff of 1e-2. The tendency of ASH1L and XPC, under non-UV conditions, to be constitutively bound to chromatin means that the ASH-CTD and XPC ChIP-seq data are not "peaky"; for this reason we used XPC broad peaks only to verify replicability with IDR, and used only continuous XPC ChIP-seq signal in downstream analyses. For comparison of ASH-CTD UV-induced binding sites with CPDs, we used the ASH-CTD ChIP-seq signal derived from the top quartile of robust (that is, shared across at least two of three triplicates) peaks at each timepoint, excluding 3H peaks that overlapped with non-UV robust peaks so as to find the highest confidence set of UV-only binding sites. For H3K4me3 ChIP-seq, 13-29% of narrow peaks for each replicate had at least 5-fold enrichment. Specifically: 18618/91762 narrow peaks for WT noUV Rep1 were at least 5-fold enrichment, 15673/111951 (WT noUV Rep2), 22233/122482 (WT noUV Rep3), 22675/111335 (WT 1H Rep1), 24267/106413 (WT 1H Rep2), 23479/142688 (WT 1H Rep3), 26486/137372 (WT 3H Rep1), 25375/119986 (WT 3H Rep2), 26099/141258 (WT 3H Rep3), 16448/110357 (AL noUV Rep1), 14860/96779 (AL noUV Rep2), 16905/125323 (AL noUV Rep3), 21018/72984 (AL 1H Rep1), 22725/120024 (AL 1H Rep2), 21569/126467 (AL 1H Rep3), 16490/123361 (AL 3H Rep1), 18400/94366 (AL 3H Rep2), 19473/120237 (AL 3H Rep3). For H3K36me2 and ASH1L-CTD ChIP-seq, both of which had broad peak distributions, less than 1% of broad peaks had above 5-fold enrichment. Between 84-96% of H3K4me3 narrow peaks had -log10 q-values of 1.3 or higher, indicating an FDR of at least 5%. Between 7-11% of H3K36me2 broad peaks and less than 1% of ASH-CTD broad peaks for each replicate had -log10 q-values of 1.3 or higher. To further ensure quality, only robust peaks overlapping across a majority (at least 2 of H3K4me3 biological triplicates; both of H3K36me2 duplicates) were used in downstream analyses, and the top quartile of robust peaks (when rank ordered by peak height) were further sub-selected and their behavior compared with the full set of robust peaks, as in Fig. 3a,b and Supplementary Figures S5a and S6a-b.

Software

Sequencing reads were provided by the Functional Genomics Center Zurich as single-end FASTQ files, with top-up reads for the H3K36me2 experiment provided as paired-end FASTQ files. Raw reads downloaded from the Genomics Center server were trimmed to remove reads shorter than 99 base pairs and crop reads exceeding 101 bp (H3K36me2 supplemental paired-end reads were cropped to 149-151 bp) (TrimGalore v.0.6.5), then aligned (bwa mem v.0.7.17-r1188) to the human reference genome build GCA hg38 lacking chrY but including non-canonical contigs. Alignments were filtered to exclude reads with mapping quality scores below 30 (bedtools v.2.29.2), PCR and optical duplicates (Picard MarkDuplicates, v.2.23.8), ENCODE-blacklisted regions, non-canonical contigs, and unmapped or secondary alignments (samtools v.1.7). Filtered alignments were used to call peaks (macs2 v.2.2.7.1 with default parameters) and to calculate fragment extension size using custom R scripts (R v.4.0.3)72,73. Peaks were called using a p-value cutoff of 1e-2. Only robust peaks found in a majority of biological replicates were used in downstream analyses. Fold enrichment tracks were generated of IP signal over input control (for H3K4me3 and H3K36me2), over IP signal from XPC–/– cells (for XPC), or over IP signal from ASH1L knockout cells transfected with empty vector (for ASH1L-CTD).

Flow Cytometry

Plots

Confirm that:

☒ The axis labels state the marker and fluorochrome used (e.g. CD4-FITC).

☒ The axis scales are clearly visible. Include numbers along axes only for bottom left plot of group (a 'group' is an analysis of identical markers).

☒ All plots are contour plots with outliers or pseudocolor plots.

☒ A numerical value for number of cells or percentage (with statistics) is provided.

Methodology

Sample preparation

U2OS cells were synchronized in 5 µM L-mimosine for 24 h. After UV-C irradiation, the cells were left to recover for 6 or 18 h

Sample preparation	with the addition of 10 µM EdU for the final 30-min incubation. The cells were collected and fixed in 4% (w/v) paraformaldehyde (pH 8.0) for 15 min at room temperature. After permeabilization in 1x saponin buffer for 15 min at room temperature, the Click-iT reaction was performed according to the manufacturer's protocol (Invitrogen). Thereafter, samples were incubated with 0.1 mg/mL RNase A for 30 min at 37°C and the DNA was stained with 25 µg/mL propidium iodide for 5 min.
Instrument	BD LSR II Fortessa flow cytometer
Software	FlowJo v10.9
Cell population abundance	10,000 cells per sample
Gating strategy	The gating strategy is depicted in the Supplementary Figure S2

☒ Tick this box to confirm that a figure exemplifying the gating strategy is provided in the Supplementary Information.
