# Supplementary material for: ASH1L-MRG15 methyltransferase deposits H3K4me3 and FACT for damage verification in nucleotide excision repair
Source: Nat Commun. 2023 Jul 1;14:3892. doi: 10.1038/s41467-023-39635-7 (PMC10314917; doi:10.1038/s41467-023-39635-7)

Supplementary data
Western blot membrane originals

Figure 1b

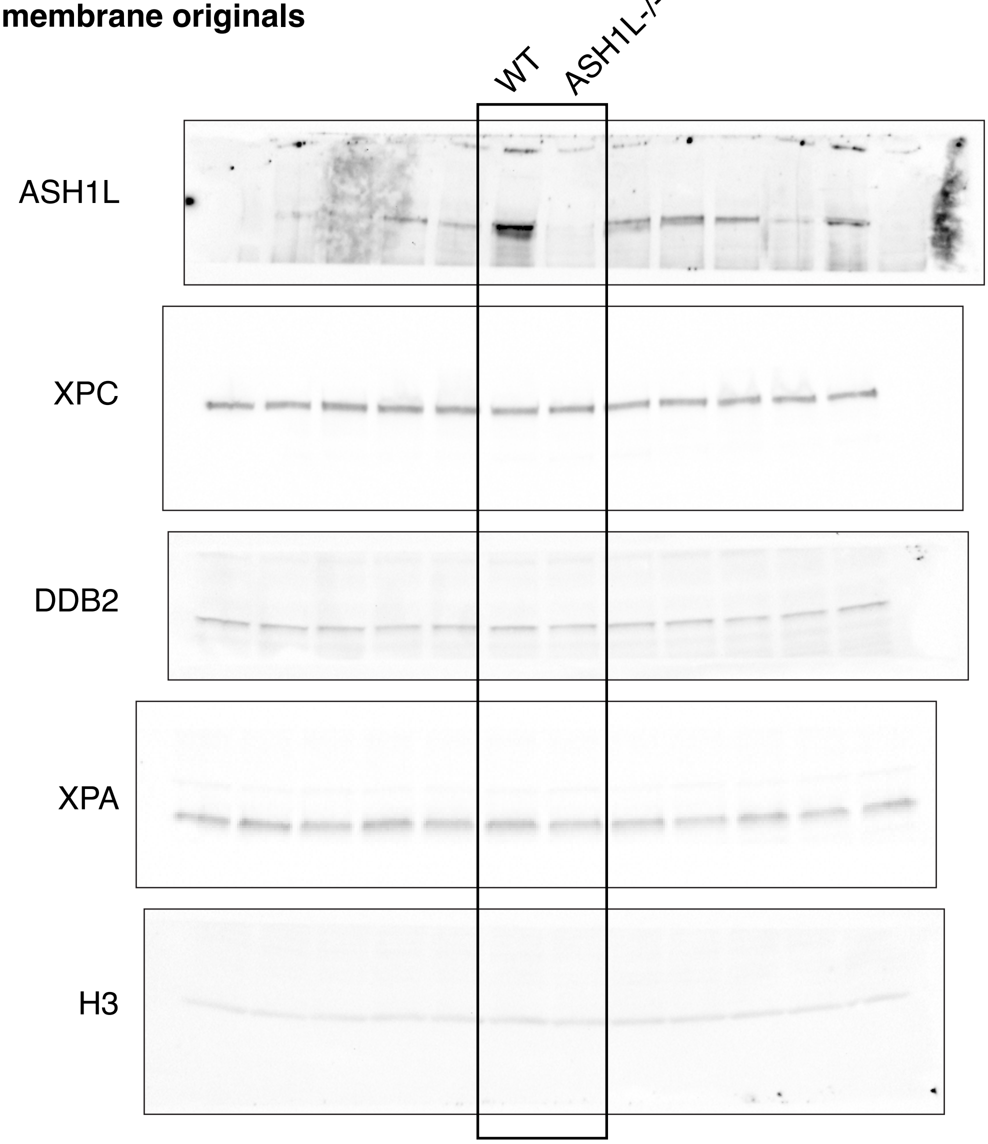

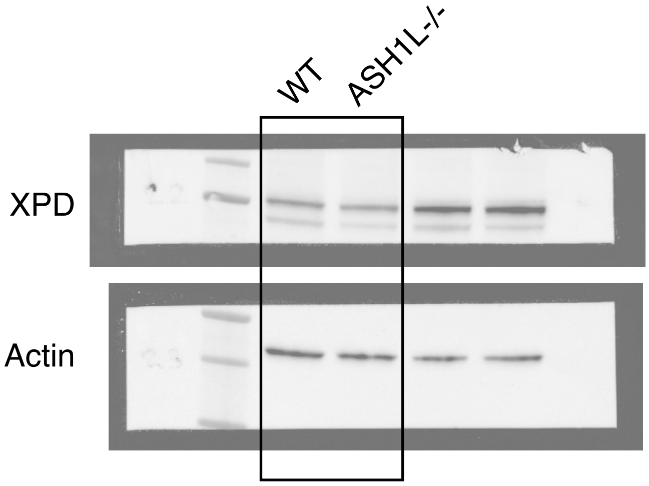

Supplementary data
Western blot membrane originals

Figure 2b

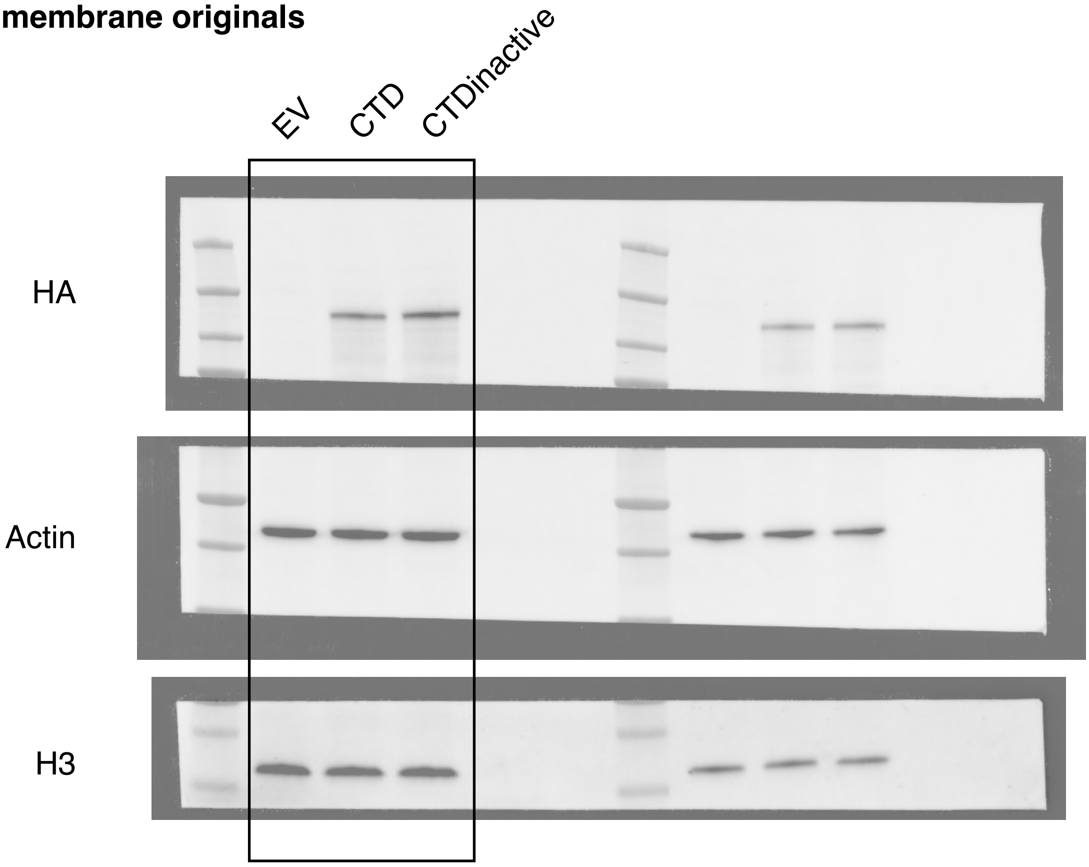

Supplementary data
Western blot membrane originals

Supplementary Figure 3c

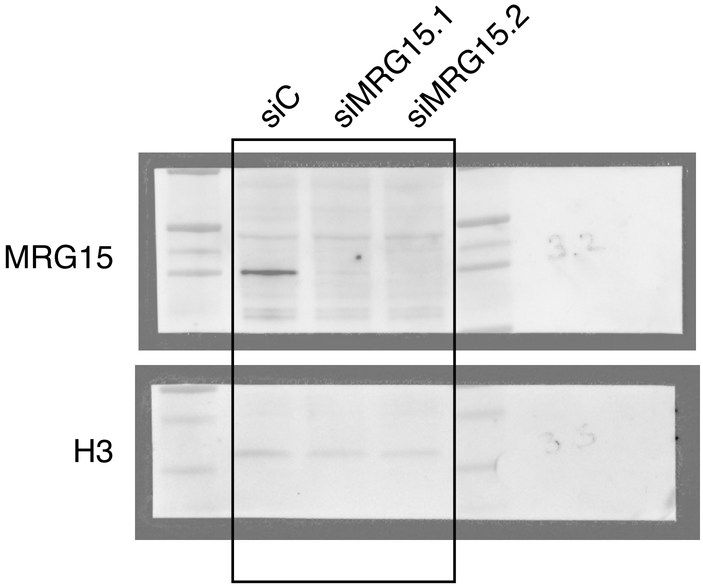

Supplementary Figure 3d

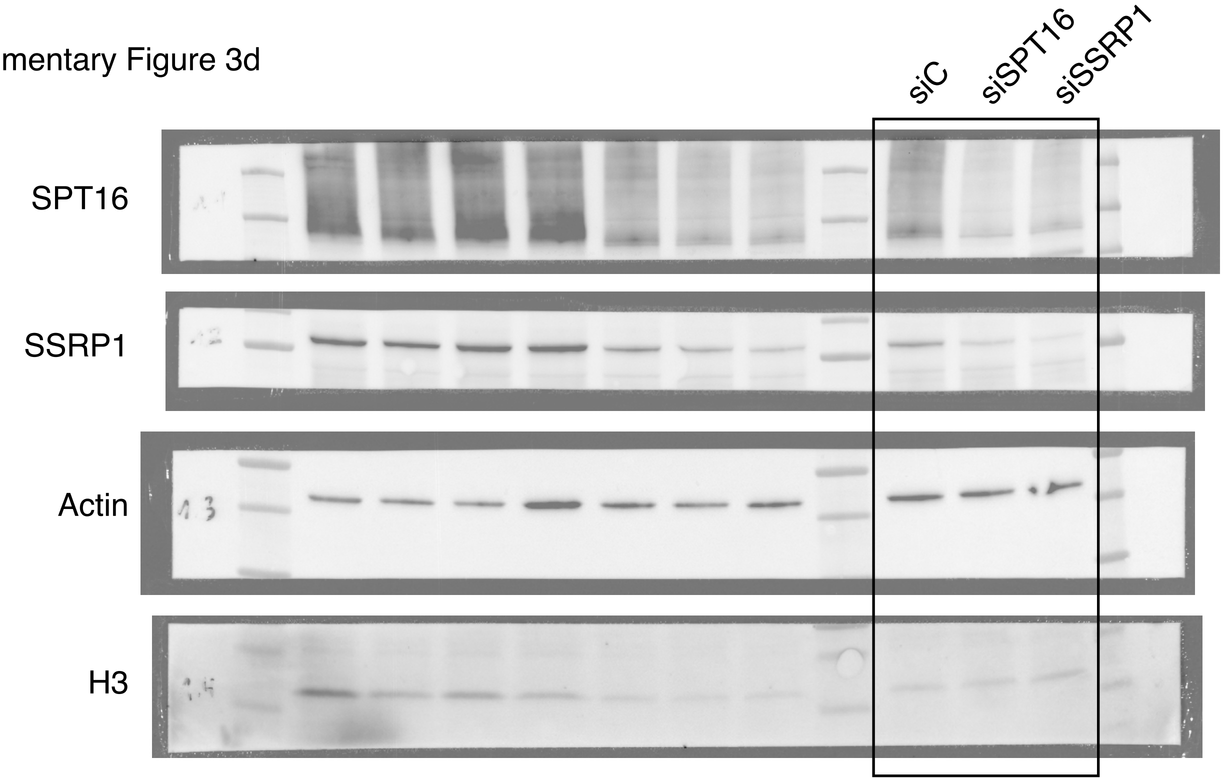

Supplementary data
Western blot membrane originals

Supplementary Figure 3i

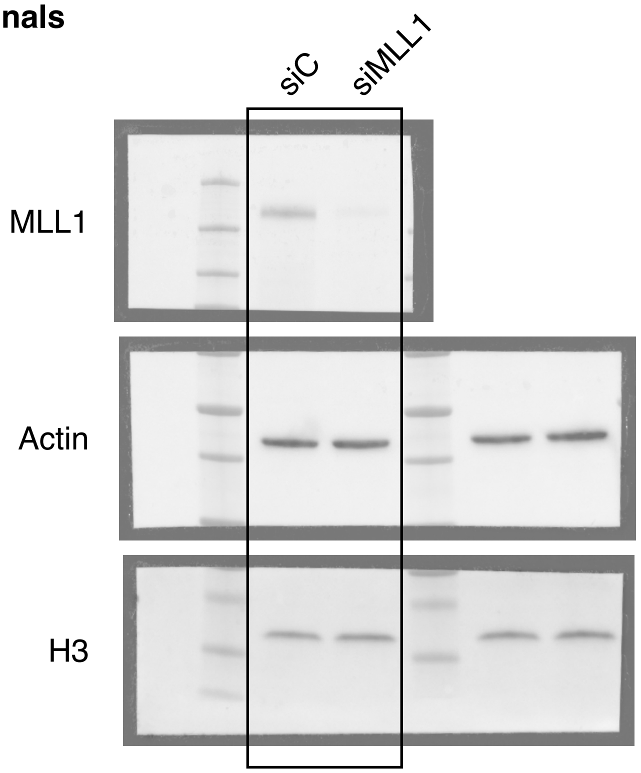

Supplementary Figure 4c

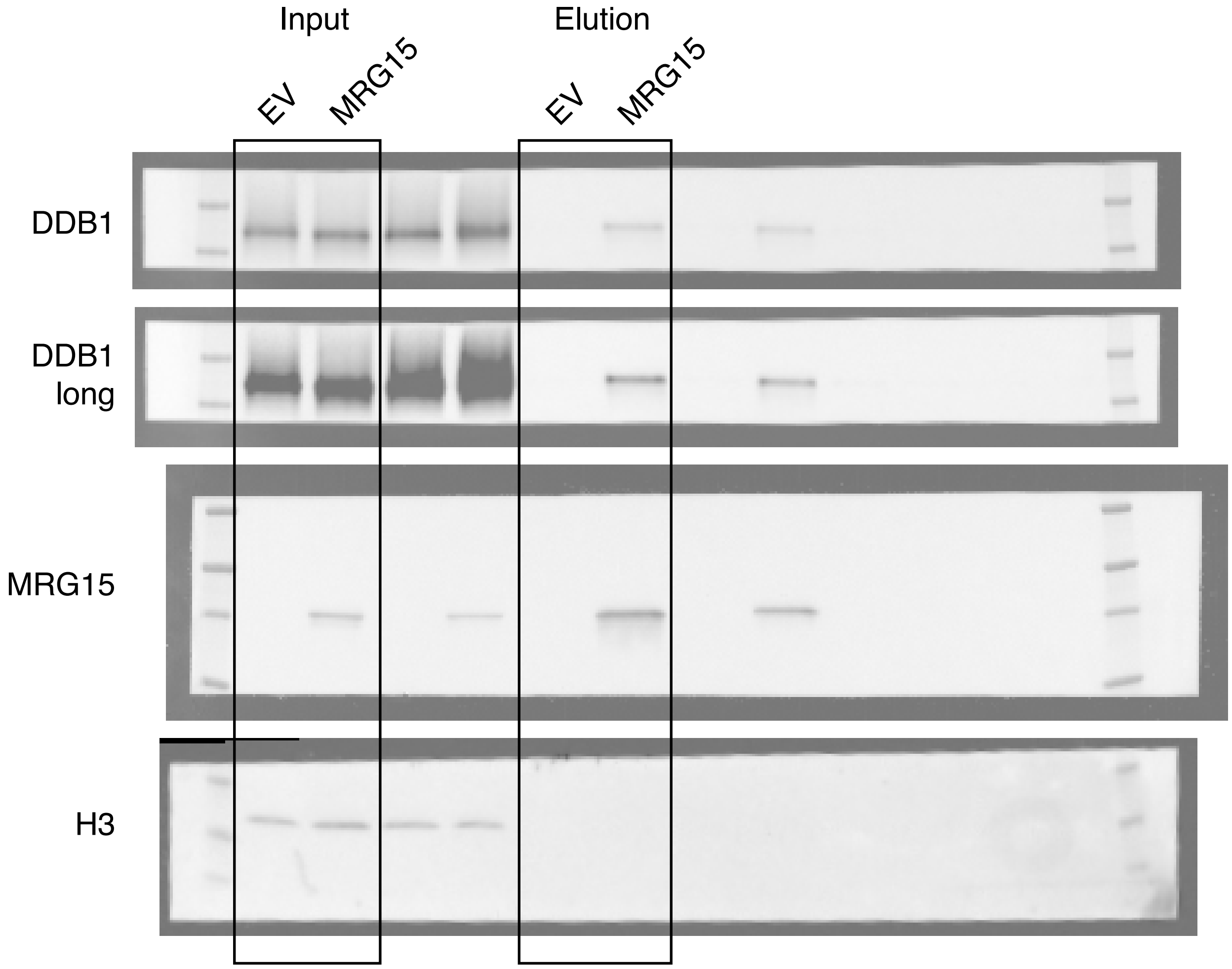

Supplementary data
Western blot membrane originals

Supplementary Figure 9a/b

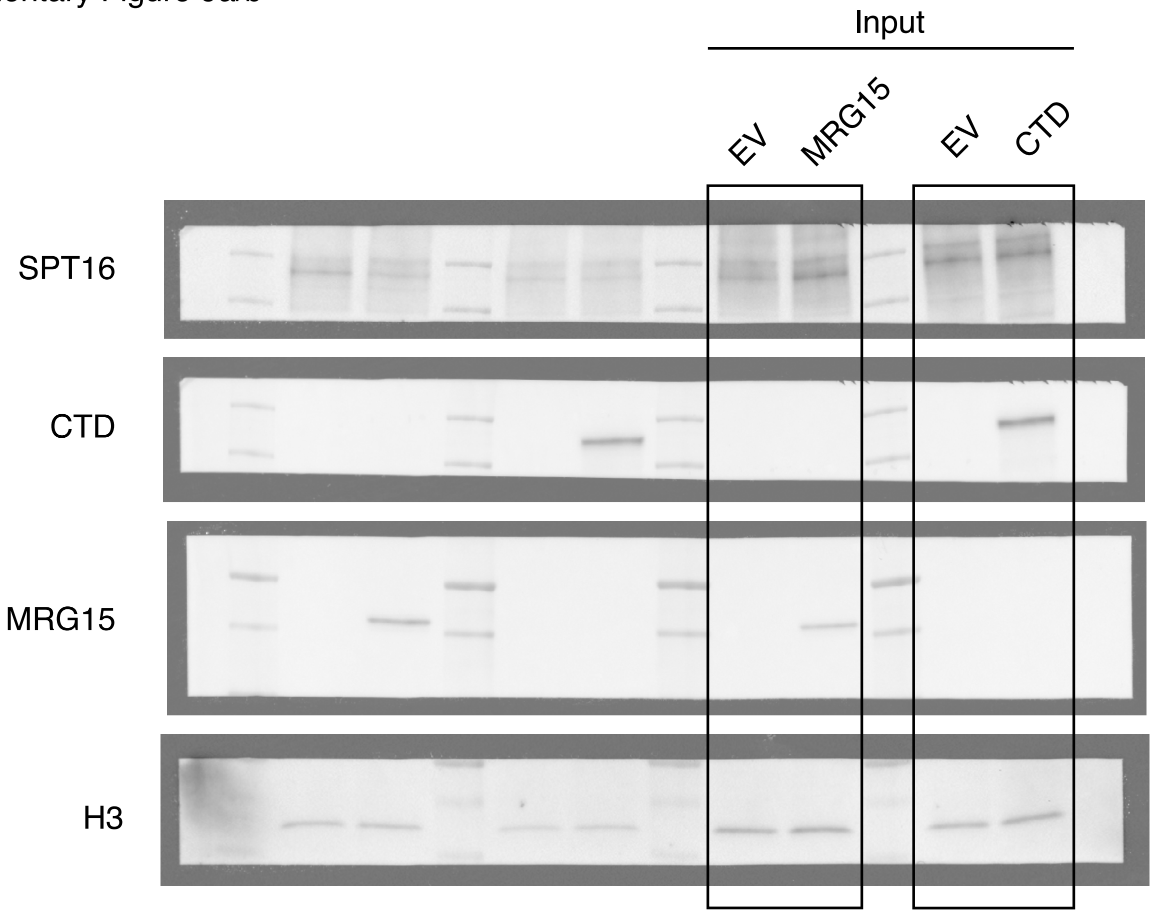

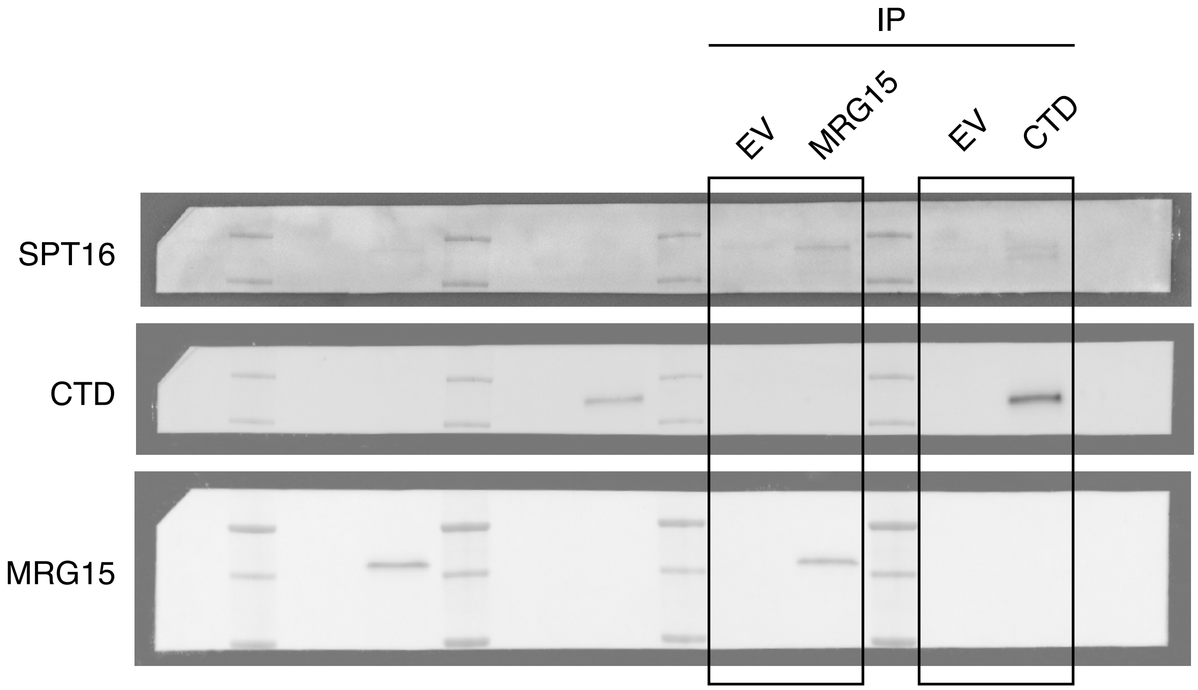

Supplement: Supplementary file 4 — Source Data [file 41467_2023_39635_MOESM4_ESM.zip › Uncropped blots.pdf]
